# Personalized Smartphone Messaging for Secondary Prevention After Percutaneous Coronary Intervention: Randomized Controlled Trial

**DOI:** 10.2196/81524

**Published:** 2026-04-23

**Authors:** Sunki Lee, Ji Bak Kim, Seung Eon Shin, Jeonghoon Ahn, Hyun-Ghang Jeong, Kyung Ho Jung, Younghoon Song, Eung Ju Kim

**Affiliations:** 1 Department of Cardiology, Korea University Guro Hospital, 148, Gurodong-Ro, Guro-gu, Seoul, 08308, Republic of Korea, 1 82-2-2626-3022; 2 Division of Cardiology, Department of Internal Medicine, Hanil General Hospital, Seoul, Republic of Korea; 3 Department of Health Convergence, Ewha Womans University, Seoul, Republic of Korea; 4 Department of Psychiatry, Korea University Guro Hospital, Korea University College of Medicine, Seoul, Republic of Korea; 5 Department of IT Business, Hanmi Pharmaceutical (South Korea), Seoul, Republic of Korea

**Keywords:** cardiac rehabilitation, coronary artery disease, mobile health, mHealth, smartphone app, text messaging, risk factor modification, secondary prevention

## Abstract

**Background:**

Cardiac rehabilitation improves outcomes after percutaneous coronary intervention (PCI), but participation remains suboptimal due to barriers such as distance, cost, and lack of referral. Mobile health interventions may enhance accessibility and engagement.

**Objective:**

This study evaluated the effectiveness and user acceptability of a smartphone messaging app (AnSim) in improving cardiovascular risk factors among patients who recently underwent PCI.

**Methods:**

A 2-arm randomized controlled trial was conducted at 2 Korean hospitals; 120 patients who had undergone PCI within the prior month were randomized (1:1) to receive AnSim plus usual care (intervention) or usual care alone (control). The intervention comprised personalized messages tailored by behavioral stage and risk profile, delivered 6 times/week for 6 months. Outcomes were assessed at baseline, 6 months, and 9 months. The primary outcome was change in blood pressure (BP) at 6 and 9 months. Secondary outcomes were lipid profiles, hemoglobin A_1c_, BMI, smoking status, physical activity, and achievement of guideline-recommended risk-factor targets. User acceptability was assessed by questionnaire.

**Results:**

Overall, 115 (95.8%; intervention: n=58, control: n=57) participants completed follow-up at 6 months and 110 (91.7%; intervention: n=54, control: n=56) at 9 months. At 6 months, systolic BP was 124.9 (SD 16.7) mmHg in the intervention group and 124.8 (SD 13.1) mmHg in the control group (between-group difference 0.1 mmHg, 95% CI −5.4 to 5.6), and diastolic BP was 80.2 (SD 11.4) mmHg vs 78.4 (SD 10.6) mmHg (difference 1.8 mmHg, 95% CI −2.3 to 5.9). At 9 months, systolic BP was 126 (SD 14.2) mmHg vs 128 (SD 15.4) mmHg (difference −2 mmHg, 95% CI −7.6 to 3.6), and diastolic BP was 80.8 (SD 13.2) mmHg vs 81.3 (SD 12.0) mmHg (difference −0.5 mmHg, 95% CI −5.3 to 4.3). No significant between-group differences were observed in secondary outcomes. In post hoc analyses, participants with above-median message reading were more likely to achieve ≥4 of 5 guideline-recommended risk-factor targets at 9 months (69% vs 19.2%; relative risk 3.59, 95% CI 1.57‐8.18; *P*<.001) and logged more diary entries (mean difference 203.4 entries, 95% CI 79.3‐327.5; *P*=.001). BP responders at 9 months read messages on more days than nonresponders (110.3 vs 83.3 d; mean difference 27 d, 95% CI 4.6‐49.3; *P*=.02). Among survey respondents (54/60, 90%), 87% (47/54) found the messages helpful and 81.4% (44/54) wished to continue. Two events occurred in the control group (*P*=.50).

**Conclusions:**

This trial found no between-group improvement in BP with low-intensity personalized messaging compared to an attention-matched diary app, but exploratory analyses suggest engagement may be a key determinant of potential benefit. Unlike comprehensive app-based cardiac rehabilitation programs, AnSim provides scalable behavioral reinforcement that can be integrated into post-PCI follow-up with minimal burden. These findings support future pragmatic trials optimizing engagement strategies and evaluating longer-term clinical and implementation outcomes.

## Introduction

Cardiovascular (CV) disease, including coronary artery disease (CAD) and stroke, remains a leading cause of morbidity and mortality worldwide [1,2]. Even after successful percutaneous coronary intervention (PCI), patients with CAD remain at substantial risk of recurrent myocardial infarction, rehospitalization, and premature death, contributing to ongoing health care use and costs [3,4]. Primary prevention aims to prevent the first occurrence of CV events, whereas secondary prevention focuses on reducing recurrence and improving outcomes in patients with established CV disease. In the post-PCI setting, secondary prevention strategies, such as cardiac rehabilitation (CR), are important for reducing recurrence and improving patient outcomes.

CR is a comprehensive, guideline-based intervention designed to promote healthy lifestyles and address modifiable CV risk factors through education, exercise training, dietary counseling, and stress management [5]. Participation in CR has been associated with significantly reduced CV morbidity and mortality and improved quality of life, with evidence supporting its cost-effectiveness [6-8]. However, despite its clear benefits, global CR participation remains low, reported in 20% to 50% of patients [9]. This reflects both limited availability and access in some settings and low uptake or sustained adherence even when CR is offered, and these factors may vary by health system and geography [10,11]. Patients with multiple CV risk factors or low health literacy, a particularly at-risk population, are among the least likely to attend [12] and adhere to CR [13].

Barriers to CR participation and sustained secondary prevention are multifactorial. System-level barriers include limited referral and program capacity, geographic inaccessibility, costs, and insurance constraints [14,15], whereas patient-level barriers include limited knowledge of CR, low perceived benefit or motivation, competing demands, physical limitations, and challenges maintaining health behaviors over time [16,17]. Accordingly, scalable interventions that reinforce education, motivation, and self-management outside clinic visits may be valuable, particularly when structured CR participation is limited. Mobile communication technologies offer opportunities to deliver low-cost, individualized support at scale. SMS text messaging has been used for health behavior interventions and, in patients with CAD, has been associated with improvements in medication adherence and risk-factor management in several studies [18,19]. As smartphone capabilities have expanded, mobile apps can deliver tailored content and support self-monitoring, though evidence remains mixed regarding their clinical impact on sustained risk-factor control beyond usual care [20].

Although prior digital secondary prevention and CR interventions have shown promise, many rely on more resource-intensive components such as structured exercise modules, real-time monitoring, or intensive coaching, which may limit scalability in routine post-PCI care [21,22]. Evidence remains limited on whether a lightweight, theory-informed, personalized messaging intervention can improve risk-factor control while remaining feasible for integration into usual follow-up workflows.

In this context, we developed AnSim, a smartphone-based, patient-specific messaging program designed to support post-PCI secondary prevention through personalized educational and motivational messages, with optional self-monitoring features. AnSim was not intended to deliver comprehensive CR but rather to provide scalable reinforcement of guideline-recommended behaviors and risk-factor management following PCI. Our primary objective was to determine whether this personalized messaging intervention improves blood pressure control compared to usual care. We also explored whether higher levels of patient engagement with the app would be positively associated with the attainment of guideline-recommended CV risk-factor targets.

## Methods

### Trial Design

The clinical trial was designed as a single-blinded, 2-arm randomized controlled trial with a total follow-up period of 9 months, comprising 6 months of intervention and 3 months of additional observation. The trial was conducted at 2 sites in Korea: a secondary general hospital (Sejong General Hospital) and a tertiary academic hospital (Korea University Guro Hospital). The trial was registered at Clinical Research Information Service (CRIS) (KCT0002361) [23]. Registration occurred prospectively on August 10, 2016, and the first participant was enrolled on December 17, 2016. This study is reported in accordance with the CONSORT (Consolidated Standards of Reporting Trials) 2025 statement and the CONSORT-EHEALTH (Consolidated Standards of Reporting Trials of Electronic and Mobile Health Applications and Online Telehealth) checklist (Checklist 1) for electronic health and mobile health randomized trials [24,25], and the completed CONSORT 2025 checklist is provided in Checklist 2.

#### Participants and Setting

Eligible participants were adults (≥18 y) who had undergone PCI within the previous 1 month and were able to use a smartphone. Patients with cognitive dysfunction (eg, stroke or dementia) or significant psychiatric disorders (eg, anxiety, depression) were excluded. PCI was performed in patients with significant anatomic stenosis (>50% in the left main or >70% in non–left main coronary arteries) or physiologic ischemia (fractional flow reserve <0.80) and included cases of ST-segment elevation myocardial infarction, non–ST elevation acute coronary syndrome, unstable angina, and stable angina. All patients received guideline-directed medical therapy. Exclusion criteria included inability to use a smartphone, nonfluency in Korean, prior coronary artery bypass graft surgery, uncontrolled arrhythmia or heart failure, severe chronic obstructive pulmonary disease or asthma, end-stage renal disease requiring dialysis, or terminal malignancy.

#### Randomization and Blinding

Participants were randomly assigned to either the intervention or control group using a centralized, computer-generated randomization table with a 1:1 allocation ratio and a block size of 4. To minimize performance and measurement bias, all participants were provided with a general health monitoring app (“Heart Keeper”) developed by the Korean Society of Cardiology. To support participant blinding, the Heart Keeper app was installed for both groups; Heart Keeper functioned as a simple diary-style tool for recording clinical information and did not deliver structured behavior-change messaging or coaching. The intervention group additionally received AnSim. A comparison between the 2 apps is listed in Table S1 in Multimedia Appendix 1. Due to the nature of the intervention (personalized smartphone messages), participants could not be fully blinded after group allocation. Blinding was maintained by ensuring that outcome assessors and investigators were unaware of group allocation. Research nurses trained in allocation concealment and participant instruction managed all app installations. Participants were instructed not to disclose their app usage to study staff during follow-up visits.

### Ethical Considerations

The study protocol was approved by the Institutional Review Board of Korea University Guro Hospital (approval number MD16037-002). All participants provided written informed consent prior to enrollment; the consent process included permission for use of collected data for the prespecified analyses reported in this manuscript. Study data were deidentified and stored on encrypted, access-restricted institutional servers, and only authorized study personnel had access. Participants were provided with contact details to report any privacy-related concerns. Participants did not receive financial compensation for their participation in this study. No identifiable images of individual participants or users are included in the manuscript or supplementary materials.

### Interventions

A detailed description of the AnSim app development and usability testing has been published previously [26]. Briefly, AnSim was developed through multidisciplinary collaboration (cardiology, behavioral science, nursing, pharmacy, nutrition, rehabilitation, and app development), informed by focus group interviews, and implemented as a structured message bank spanning 5 domains (CV health/medications, nutrition, physical activity, stress management, and smoking cessation). Messages were tailored according to simplified transtheoretical model stages and mapped to 26 behavior change techniques [27]; iterative refinement incorporated expert review and feedback from potential users, followed by pilot testing of the delivery system.

#### Intervention Development: Focus Group Interviews

To capture patient needs and inform message development, a focus group interview was conducted with 8 patients who had previously undergone PCI and used a smartphone. Participants were purposefully sampled to reflect a diversity of age, sex, and education level. The subject of the interview consisted of five categories: (1) degree of use of the smartphone app, (2) exercise, (3) nutrition, (4) stress management, and (5) knowledge about CAD and prevention. Interviews were recorded with patient consent and textualized in verbatim form, and qualitative analysis was performed using NVivo (QSR International).

#### Intervention Development: Message Drafting and Refinement

Based on insights from the focus group interview, health experts drafted messages in five thematic categories: (1) general CV health and medications, (2) nutrition, (3) physical activity, (4) stress management, and (5) smoking cessation. Each message contained 40 to 140 Korean characters and was adapted from international guidelines and official educational resources from CV health–related academic societies.

A total of 450 messages (90 per category) was initially developed and reviewed for clinical accuracy, literacy level, and relevance. Subsequently, 200 potential users evaluated each message using a 5-point Likert scale for readability and usefulness. Most messages received favorable scores (mean 3.95, SD 0.39 for readability and mean 3.91, SD 0.39 for usefulness), and 98% (n=441) scored ≥3 on both metrics. Messages were refined accordingly, and examples are provided in Table S2 in Multimedia Appendix 1.

#### Intervention Development: Theoretical Framework

The messaging content was informed by 26 established behavior change techniques derived from models such as the information-motivation-behavioral skills model, theory of planned behavior, social cognitive theory, and operant conditioning [27]. Multimedia elements (eg, video clips, illustrations) were integrated when possible to support learning and reinforce motivation.

To match message content to individual readiness for change, we applied a simplified transtheoretical model with three behavioral stages: (1) precontemplation, (2) contemplation and preparation, and (3) action and maintenance [26]. According to these simplified steps, behavior change techniques were categorized, and 30 messages were developed for each. Each patient’s behavioral stage for each message category was reassessed monthly through the app, and messaging was adapted accordingly.

#### Intervention Delivery

Participants in the intervention group received 6 messages per week over a 24-week period. Messages were sent randomly at chosen times (9:00 AM, 12:00 PM, or 3:00 PM) from Monday through Saturday. The content was personalized based on baseline characteristics (eg, presence of diabetes, smoking status) and behavioral stage. Messages were drawn from the 5 categories mentioned above, with 5 standard messages per week plus 1 additional message targeting an individual’s weakest behavior domain (eg, nutrition or smoking cessation). Messages were never repeated for the same individual. Both the AnSim and Heart Keeper apps allowed optional tracking of health metrics (eg, blood pressure, glucose, exercise, diet). To support participant blinding, Heart Keeper was installed in both groups and functioned as a diary-style app for recording clinical information; it did not deliver structured behavior-change messaging, coaching, or interactive rehabilitation modules. All participants received brief onboarding at enrollment. To enhance engagement, participants in the intervention group also received weekly feedback messages based on their logged health data, sent by a designated health care provider. No changes in app functionality, message content, or technical downtimes occurred during the intervention period.

### Outcomes

#### Primary and Secondary Outcomes

Participants were assessed at baseline, 6 months, and 9 months. The primary outcome was the change in blood pressure, measured after 10 minutes of seated rest using a standardized automatic device (HEM-7080IT, OMRON Healthcare). Secondary outcomes were lipid profiles (total cholesterol, low-density lipoprotein cholesterol [LDL-C], high-density lipoprotein cholesterol, triglycerides), hemoglobin A_1c_ (HbA_1c_), 6-minute walk distance, self-reported smoking status, and venous carboxyhemoglobin levels as a biochemical indicator of smoking [28]. Exploratory (post hoc) analyses were performed within the intervention group to examine associations between engagement metrics and risk-factor target achievement. Participants were classified as blood pressure responders if their blood pressure was less than or equal to 130/80 mmHg at 9 months. These exploratory analyses were considered hypothesis-generating.

#### Process Outcomes

The process evaluation comprised of (1) objective app usage metrics (eg, message delivery success, message reading frequency, and health data entry frequency) and (2) app acceptability and usability, assessed at 6 months using a postintervention self-report questionnaire evaluating satisfaction with and perceived utility of the messages on a 5-point Likert scale.

#### Sample Size

The sample size was calculated based on the TEXT-ME (Tobacco, Exercise and Diet Messages) study [18], which reported a 6% reduction in systolic blood pressure (SBP; approximately 8 mmHg difference) in the intervention group compared to the control group. Based on these parameters *(*α=.05, β=.20, power=80%), the minimum required sample size was calculated as 121 using MedCalc (version 16.2.1; MedCalc Software). Although the calculated target was 121, this study enrolled a total of 120 patients (60 per group), which approximates the required power. To evaluate the missingness mechanism across prespecified continuous outcomes (SBP/diastolic blood pressure [DBP], BMI, lipid profile, and HbA_1c_ measured at baseline, 6 mo, and 9 mo), we performed the Little test for missing completely at random (MCAR).

### Statistical Analysis

All analyses were performed on an intention-to-treat basis using SPSS version 20.0 (IBM Corp). Continuous variables are presented as mean (SD); categorical variables are reported as n (%). Normality was assessed using the Shapiro-Wilk test and variance homogeneity by *F* test. Between-group comparisons for continuous variables were performed using independent-samples *t* tests (2-tailed). Categorical variables were compared using Pearson chi-square or Fisher exact tests, as appropriate. To examine group-by-time interactions for repeated measures, 2-way mixed ANOVA was used. Relative risks and 95% CIs for outcomes were estimated using Cox proportional hazards models. All statistical tests were 2-sided, and *P*<.05 was considered statistically significant.

## Results

### Participant Flow and Recruitment

**Figure 1.**
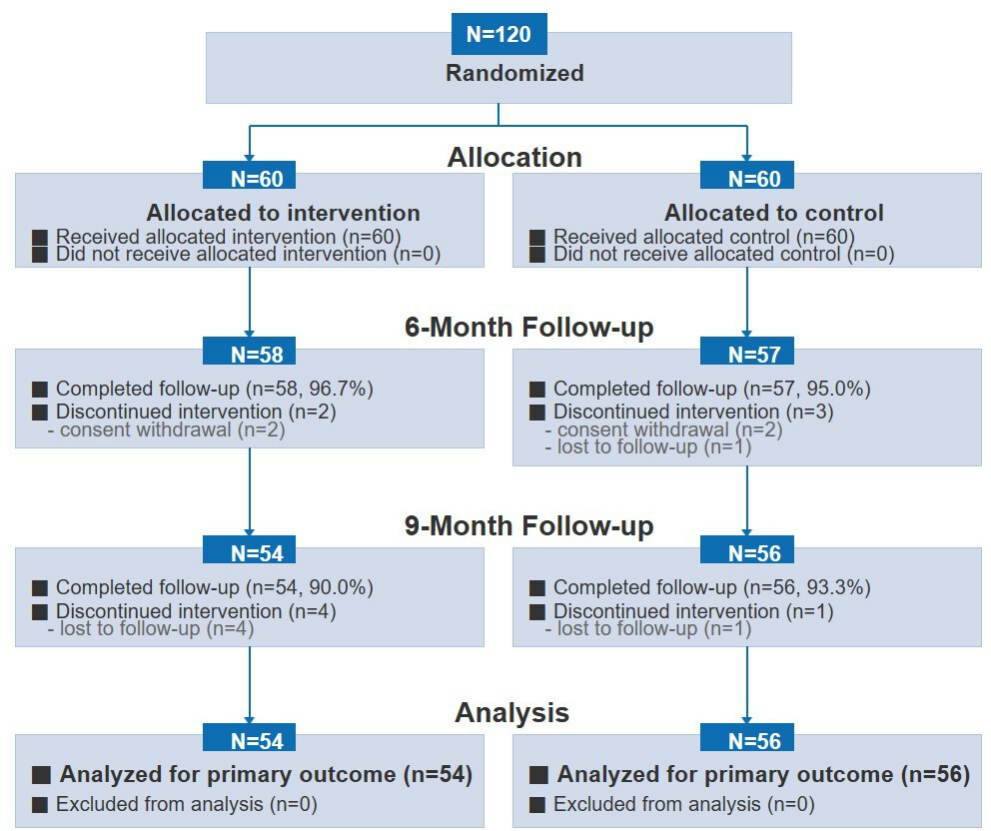
CONSORT (Consolidated Standards of Reporting Trials) flow diagram of participant enrollment, randomization, follow-up, and analysis. A total of 120 patients were randomized to the intervention group (n=60) or the control group (n=60), and all received the allocated condition. Follow-up completion was 96.7% (58/60) in the intervention group and 95% (57/60) in the control group at 6 months, and 90% (54/60) and 93.3% (56/60), respectively, at 9 months. Reasons for noncompletion at 6 months and additional attrition between 6 and 9 months are shown. The number of patients assessed for eligibility before consent was not prospectively recorded in the original trial dataset and, therefore, could not be reported.

A total of 120 patients were enrolled between December 2016 and April 2018 and randomized into the intervention group (n=60) and the control group (n=60). Participants were recruited consecutively from the participating PCI clinics during the enrollment period (ie, a convenience sample of eligible patients presenting to the sites), and randomization occurred after written informed consent and baseline assessment. Follow-up completion rates were high: 96.7% (58/60) in the intervention group and 95% (57/60) in the control group at 6 months, and 90% (54/60) and 93.3% (56/60), respectively, at 9 months. By 6 months, 2 participants in the intervention group and 3 in the control group did not complete follow-up. Between 6 and 9 months, additional attrition occurred in 4 and 1 participants, respectively. Overall, reasons for attrition included consent withdrawal (2 in each group) and loss to follow-up (4 in the intervention group, 2 in the control group) (Figure 1).

### Baseline Data

Baseline characteristics are shown in Table 1. The mean age of participants was 58.5 (8.8) years, and 84.2% (101/120) were male. However, the prevalence of diabetes mellitus was significantly lower in the intervention group (21.7% vs 41.7%, *P*=.02), resulting in a lower baseline HbA_1c_ (6.13% vs 6.60%, *P*=.048). No significant differences were observed in baseline blood pressure, lipid profiles, BMI, medication usage, or proportion achieving guideline-recommended goals. The medications prescribed in each group at the time of discharge after index coronary intervention were similar. Baseline clinical variables were nearly complete (mean missingness 0.83%). Missingness increased at follow-up (mean missingness 12.40% at 6 mo and 23.14% at 9 mo across the prespecified continuous outcomes) and was broadly similar between groups (eg, SBP at 9 mo: 16.7% vs 20%; HbA_1c_ at 9 mo: 23.3% vs 30%). While the Little MCAR test across 24 continuous clinical variables did not reject the MCAR assumption (*χ*²_137_=152.83, *P*=.17), indicating insufficient evidence of a systematic missingness pattern, this result suggests rather than definitively proves that the data are missing completely at random.

**Table 1.** Baseline characteristics.

Characteristics	Intervention (n=60)	Control (n=60)	Total (N=120)	*P* value^a^
Demographics				
Age (y), mean (SD)	59.1 (8.2)	57.2 (7.9)	58.5 (8.8)	.22
Male sex, n (%)	51 (85)	50 (83.3)	101 (84.2)	.80
Clinical data, mean (SD)				
Height (cm)	166.4 (7.6)	166.1 (7.8)	166.3 (7.7)	.84
Weight (kg)	71.7 (11.4)	70.3 (10.8)	71 (11.1)	.50
BMI (kg/m²)	25.8 (3)	25.4 (3)	25.6 (3)	.49
Clinical risk factors				
Acute coronary syndrome, n (%)	35 (58.3)	40 (66.7)	75 (62.5)	.35
Hypertension, n (%)	31 (51.7)	36 (60)	67 (55.8)	.36
Diabetes mellitus, n (%)	13 (21.7)	25 (41.7)	38 (31.7)	.02
Dyslipidemia, n (%)	28 (46.7)	30 (50)	58 (48.3)	.72
Heart failure, n (%)	2 (3.3)	2 (3.3)	4 (3.3)	>.99
Stroke, n (%)	0 (0)	0 (0)	0 (0)	NA^b^
Cholesterol (mg/dL), mean (SD)				
Total cholesterol	170.4 (43.4)	175 (47.8)	172.7 (45.5)	.58
LDL-C^c^	97.7 (37)	102.6 (35.4)	100.1 (36.1)	.46
HDL-C^d^	44.3 (12.3)	45.1 (10.1)	44.7 (11.2)	.69
Triglycerides	160.73 (137.8)	162.7 (203.7)	161.7 (173.2)	.95
Systolic BP^e^ (mmHg), mean (SD)	124.9 (16.1)	124.8 (15)	124.8 (15.5)	.97
Diastolic BP (mmHg), mean (SD)	73.2 (9.4)	73.1 (9.3)	73.1 (9.3)	.99
HbA_1c_^f^ (%), mean (SD)	6.13 (1)	6.6 (1.4)	6.4 (1.2)	.048
Carboxyhemoglobin (%), mean (SD)	0.6 (0.6)	0.9 (1.2)	0.7 (1)	.15
Ejection fraction (%), mean (SD)	59.1 (8.2)	57.2 (7.9)	58.1 (8.1)	.22
6-min walk distance (m), mean (SD)	421.7 (126.9)	404.3 (123)	413 (124.7)	.45
Current smoker, n (%)	17 (28.3)	17 (28.3)	34 (28.3)	>.99
Guideline, n (%)				
LDL-C <70 mg/dL	18 (30)	12 (20)	30 (25)	.21
BP <140/90 mmHg	51 (85)	47 (78.3)	98 (81.7)	.35
Regular exercise	26 (43.3)	23 (38.3)	49 (40.8)	.58
Nonsmoker	43 (71.7)	43 (71.7)	86 (71.7)	>.99
BMI <25 kg/m^2^	22 (36.7)	26 (43.3)	48 (40)	.46
Achieving 5 of 5	0 (0)	1 (1.7)	1 (0.8)	.32
Achieving 4 of 5	17 (28.3)	10 (16.7)	27 (22.5)	.13
Medications, n (%)				
Dual antiplatelet therapy	59 (98.3)	60 (100)	119 (99.2)	>.99
Renin-angiotensin system blocker	34 (56.7)	39 (65)	73 (60.8)	.35
Beta-blocker	37 (61.7)	41 (68.3)	78 (65)	.44
Statin	56 (95.8)	59 (98.3)	115 (95.8)	.36
All 4 medication classes	21 (35)	32 (53.3)	53 (43.2)	.04

a Independent *t* test between groups.

b N/A: not available.

c LDL-C: low-density lipoprotein cholesterol.

d HDL-C: high-density lipoprotein cholesterol.

e BP: blood pressure.

f HbA_1c_: hemoglobin A_1c_.

### Outcomes and Estimation

**Figure 2.**
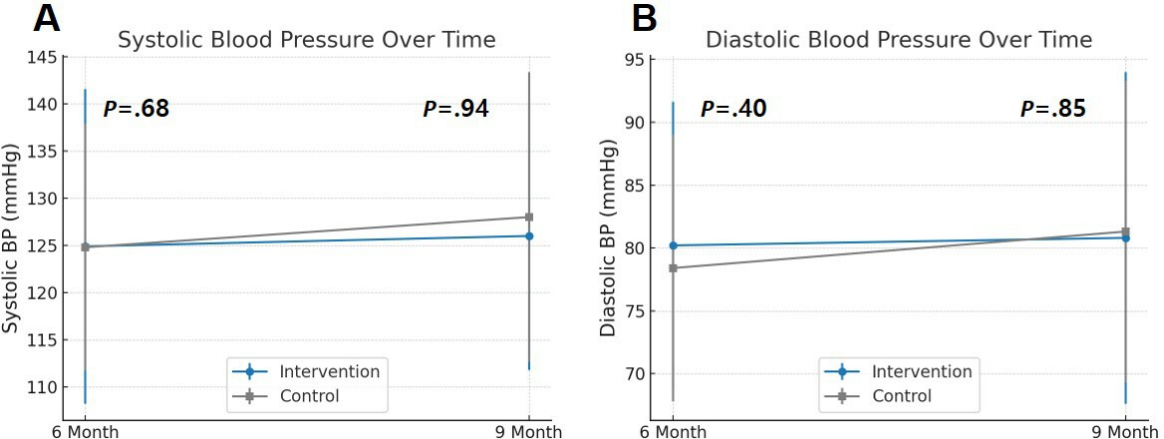
Changes in blood pressure over time between intervention and control groups. (A) Systolic blood pressure and (B) diastolic blood pressure were assessed at 6 and 9 months. Mean values and SDs are shown for both groups. Points denote group means, and error bars indicate SDs. BP: blood pressure.

Changes in blood pressure over time were not significantly different between groups at either 6 or 9 months (Figure 2; Table 2). At 9 months, mean SBP was 126 (14.2) mmHg in the intervention group vs 128 (15.4) mmHg in the control group (*P*=.94); diastolic pressure was 80.8 (13.2) mmHg vs 81.3 (12) mmHg (*P*=.85). There was no significant interaction effect between time and group for systolic (*F*_2,95_=0.029; *P*=.97) or diastolic (*F*_2,95_=0.333; *P*=.72) blood pressure. Similarly, no significant differences were observed in secondary outcomes, including lipid levels, HbA_1c_, BMI, walking distance, or smoking rates at either follow-up point. The proportion of patients achieving all 5 guideline-recommended targets (LDL-C <70 mg/dL, BP <140/90 mmHg, regular exercise, nonsmoking status, BMI <25 kg/m²) did not differ significantly between groups at 6 or 9 months (Table 3).

**Table 2.** Primary and secondary outcomes analyses at 6 and 9 months of follow-up.

Variable	Intervention (6 months: n=58; 9 months: n=54)	Control (6 months: n=57; 9 months: n=56)	*P* value^a^
Primary outcome			
Systolic BP^b^ (mmHg), mean (SD)			
6 months	124.9 (16.7)	124.8 (13.1)	.68
9 months	126 (14.2)	128 (15.4)	.94
Diastolic BP (mmHg), mean (SD)			
6 months	80.2 (11.4)	78.4 (10.6)	.40
9 months	80.8 (13.2)	81.3 (12)	.85
Secondary outcome			
Total cholesterol (mg/dL), mean (SD)			
6 months	130.1 (26.2)	136.3 (27.6)	.24
9 months	134.4 (31.5)	132.5 (20)	.74
Triglycerides (mg/dL), mean (SD)			
6 months	146.1 (97.8)	144.3 (98.5)	.93
9 months	137.9 (71.3)	144.5 (85.7)	.69
HDL-C^c^ (mg/dL), mean (SD)			
6 months	44.1 (9.7)	47.2 (11.8)	.14
9 months	46.8 (17.8)	45.3 (10.2)	.63
LDL-C^d^ (mg/dL), mean (SD)			
6 months	67 (20.6)	70.1 (21)	.45
9 months	70.5 (29.4)	69.5 (17.1)	.86
HbA_1c_^e^ (%), mean (SD)			
6 months	6 (0.7)	6.3 (0.9)	.10
9 months	6.1 (0.7)	6.3 (0.9)	.18
Body weight (kg), mean (SD)			
6 months	71 (10.8)	68.7 (9.8)	.25
9 months	72.2 (11.4)	68.8 (9.9)	.19
BMI (kg/m^2^), mean (SD)			
6 months	25.8 (2.9)	25 (3)	.29
9 months	25.8 (2.9)	25 (3)	.15
6-min walk distance (m), mean (SD)			
6 months	492.4 (87.8)	478.6 (83.9)	.43
9 months	509.6 (89.5)	498.6 (76.3)	.54
Current smoking, n (%)			
6 months	11 (20.4)	10 (19.2)	.88
9 months	9 (20.5)	10 (21.7)	.69
Carboxyhemoglobin (%), mean (SD)			
6 months	1.4 (1.6)	2.1 (2.7)	.10
9 months	1.4 (1.1)	1.4 (1.7)	.98

a Independent *t* test between groups.

b BP: blood pressure.

c HDL-C: high-density lipoprotein cholesterol.

d LDL-C: low-density lipoprotein cholesterol.

e HbA_1c_: hemoglobin A_1c_.

**Table 3.** Achievement of guideline-recommended goals at the 6 and 9 months of follow-up.

Variables	Intervention, n (%)	Control, n (%)	Relative risk (95% CI)	*P* value^a^
6 months (intervention: n=58; control: n=57)				
LDL-C^b^ <70 mg/dL	31 (53.4)	32 (56.1)	0.95 (0.68‐1.33)	.85
BP^c^ <140/90 mmHg	39 (67.2)	38 (66.7)	1.01 (0.78‐1.30)	>.99
Regular exercise	41 (70.7)	39 (68.4)	1.03 (0.81‐1.32)	.84
Nonsmoker	43 (74.1)	42 (73.7)	1.01 (0.81‐1.25)	>.99
BMI <25 kg/m^2^	22 (37.9)	31 (54.4)	0.70 (0.46‐1.05)	.093
Achieving 5 of 5	5 (8.6)	11 (19.3)	0.45 (0.17‐1.20)	.11
Achieving 4 of 5	23 (39.7)	31 (54.4)	0.73 (0.49‐1.08)	.14
9 months (intervention: n=54; control: n=56)				
LDL-C <70 mg/dL	24 (44.4)	28 (50)	0.89 (0.60‐1.32)	.57
BP <140/90 mmHg	29 (53.7)	30 (53.6)	1 (0.71‐1.42)	.99
Regular exercise	36 (66.7)	31 (55.4)	1.20 (0.89‐1.63)	.25
Nonsmoker	42 (77.8)	38 (67.9)	1.15 (0.91‐1.44)	.29
BMI <25 kg/m^2^	16 (29.6)	24 (42.9)	0.69 (0.42‐1.15)	.17
Achieving 5 of 5	6 (11.1)	9 (16.1)	0.69 (0.26‐1.81)	.58
Achieving 4 of 5	19 (35.2)	22 (39.3)	0.90 (0.55‐1.46)	.70

a Relative risk and 95% CIs were calculated with Cox proportional-hazard models.

b LDL-C: low-density lipoprotein cholesterol.

c BP: blood pressure.

### Harms

During the 9-month follow-up, 2 clinical events occurred, both in the control group (1 coronary revascularization and 2 readmissions), but the difference between groups was not significant (*P*=.50; Table S3 in Multimedia Appendix 1). No deaths, myocardial infarctions, or heart failure hospitalizations were reported in either group.

### Ancillary Analysis: Message Engagement

Since the intervention using the AnSim app was message-mediated, the efficacy of the intervention could be dependent on participant message use. Among intervention group participants, the frequency of message reading was analyzed as a marker of engagement. Patients were stratified into high (upper 50%) and low (lower 50%) message readers (Table 4). High readers accessed more than twice as many messages on average (130.5 vs 50.6 messages; *P*<.001) and had significantly greater engagement in health diary input (250.2 vs 46.8 entries; *P*=.001). Although no significant differences were observed in blood pressure or lipid levels between subgroups, a significantly larger proportion of high readers achieved at least 4 of 5 guideline-recommended goals at 9 months (69% vs 19.2%; *P*<.001).

**Table 4.** Comparison according to frequency of messages read.

Parameters	Upper 50% (6 months: n=31; 9 months: n=29)	Lower 50% (6 months: n=27; 9 months: n=26)	*P* value^a^
Baseline characteristics			
Age (y), mean (SD)	57.2 (7.6)	59.9 (10.7)	.25
Male sex, n (%)	25 (80.6)	26 (96.3)	.11
Acute coronary syndrome, n (%)	20 (64.5)	15 (55.6)	.59
Achieving ≥4 goals, n (%)	23 (74.2)	20 (74.1)	>.99
Days messages were read (d), mean (SD)	130.5 (11.4)	50.6 (35)	<.001
Health diary entries (n), mean (SD)	250.2 (325.6)	46.8 (93.5)	.001
Satisfaction with AnSim app, mean (SD)	3.7 (0.7)	3.6 (0.5)	.62
Primary outcome			
Systolic BP^b^ (mmHg), mean (SD)			
6th month	125.5 (13)	122.5 (20)	.65
9th month	123.9 (13.1)	130 (15.4)	.18
Diastolic BP (mmHg), mean (SD)			
6th month	80.3 (10.2)	80.1 (12.8)	.97
9th month	80 (11.7)	81.6 (14.9)	.22
Secondary outcome			
Total cholesterol (mg/dL), mean (SD)			
6th month	132.3 (23.7)	127.8 (29)	.27
9th month	133.7 (32.4)	135.2 (31.3)	.86
Triglycerides (mg/dL), mean (SD)			
6th month	135.6 (60.1)	157.4 (126.9)	.74
9th month	141.2 (60.2)	134.1 (83.7)	.32
HDL-C^c^ (mg/dL), mean (SD)			
6th month	45.3 (10.7)	42.8 (8.7)	.62
9th month	48.9 (22.7)	44.3 (8.7)	.85
LDL-C^d^ (mg/dL), mean (SD)			
6th month	68.7 (21.9)	65.1 (19.3)	.36
9th month	67.8 (30.9)	73.4 (28.1)	.35
HbA_1c_^e^ (%), mean (SD)			
6th month	5.9 (0.8)	6.1 (0.7)	.10
9th month	6.1 (8.3)	6.1 (0.6)	.53
Body weight (kg), mean (SD)			
6th month	71 (11.3)	70.9 (10.6)	.83
9th month	72.6 (12.1)	71.7 (10.8)	.89
BMI (kg/m^2^), mean (SD)			
6th month	25.6 (2.9)	25.4 (2.5)	.99
9th month	26 (3)	25.6 (2.8)	.70
6-min walk distance (m), mean (SD)			
6th month	483.3 (87.8)	502.7 (88.6)	.41
9th month	511.3 (70.2)	507.8 (108)	.78
Current smoking, n (%)			
6th month	3 (9.7)	8 (29.6)	.09
9th month	3 (10.3)	6 (23.1)	.28
Carboxyhemoglobin (%), mean (SD)			
6th month	1.2 (1.2)	1.6 (1.9)	.73
9th month	1.2 (1.2)	1.7 (1.1)	.046
Achieving 4 of 5, n (%)			
6th month	13 (41.9)	10 (37)	.79
9th month	15 (51.7)	4 (15.4)	.01
Achieving 5 of 5, n (%)			
6th month	3 (9.7)	2 (7.4)	>.99
9th month	5 (17.2)	1 (3.8)	.20
Achieving ≥4 of 5, n (%)			
6th month	16 (51.6)	12 (44.4)	.61
9th month	20 (69)	5 (19.2)	<.001

a Independent *t* test between groups. Data are presented as mean (SD) for continuous variables and n (%) for categorical variables.

b BP: blood pressure.

c HDL-C: high-density lipoprotein cholesterol.

d LDL-C: low-density lipoprotein cholesterol.

e HbA_1c_: hemoglobin A_1c_.

### Ancillary Analysis: Blood Pressure Responders

In a subgroup analysis, patients in the intervention group were categorized based on whether they demonstrated improvement in blood pressure by 9 months (n=23) or not (n=33). Patients were categorized as blood pressure responders if their follow-up blood pressure was less than or equal to 130/80 mmHg at 9 months. Responders had significantly higher baseline systolic and DBPs but showed greater reductions over time. At 9 months, mean SBP was lower in the responder group (121.4 vs 130.8 mmHg; *P*=.02), and DBP was similarly reduced (75.9 vs 84.3 mmHg; *P*=.03; Table 5). Responders also read significantly more messages (110.3 vs 83.3 messages, *P*=.02) and achieved better overall CV risk factor control. They were more likely to meet LDL-C targets at 6 and 9 months (*P*=.06 and .05) and maintain BP less than 140/90 mmHg at 9 months (*P*=.052). At 6 months, 73.9% of the BP-improved group met at least 4 guideline-recommended goals compared to 31.4% in the nonimproved group (*P*=.04, Table S4 in Multimedia Appendix 1). At 9 months, this pattern persisted, with 71.4% of the improved group versus 30.3% of the nonimproved group achieving at least 4 of 5 goals (*P*=.045, Table S4 in Multimedia Appendix 1).

**Table 5.** Blood pressure (BP) improvement at 9 months.

Variables	BP-improved group (baseline: n=23; 6 months: n=23; 9 months: n=21)	BP not improved group (baseline: n=37; 6 months: n=35; 9 months: n=33)	*P* value^a^
Days messages were read (d), mean (SD)	110.26 (34.4)	83.30 (49.1)	.02
Systolic BP (mmHg), mean (SD)			
Baseline	135.54 (14.23)	118.32 (13.57)	<.001
6th month	125 (16.40)	123.48 (16.81)	.74
9th month	121.35 (14.82)	130.75 (13)	.02
Delta	−14.98 (10.42)	13.93 (13.08)	<.001
Diastolic BP (mmHg), mean (SD)			
Baseline	79.23 (12.13)	69.43 (8.11)	<.001
6th month	79.36 (12.13)	80.79 (11.05)	.65
9th month	75.95 (12.30)	84.25 (12.92)	.03
Delta	−2.89 (9.43)	14.65 (13)	<.001
Age (y), mean (SD)	58.8 (7.4)	58.3 (9.8)	.82
Male sex, n (%)	21 (91.3)	30 (81.1)	.28
Acute coronary syndrome, n (%)	12 (52.2)	30 (81.1)	.35
Hypertension, n (%)	13 (56.5)	23 (62.2)	.36
Diabetes mellitus, n (%)	6 (26.1)	7 (18.9)	.02
Dyslipidemia, n (%)	10 (43.5)	18 (48.6)	.72
Heart failure, n (%)	1 (4.3)	1 (2.7)	>.99
Stroke, n (%)	0 (0)	0 (0)	N/A^b^
Total cholesterol (mg/dL)			
Baseline	172.22 (51.15)	169.19 (38.47)	.80
6th month	123.36 (22.32)	134.75 (27.78)	.12
9th month	128.70 (31.29)	138.96 (31.62)	.28
LDL-C^c^ (mg/dL), mean (SD)			
Baseline	101.48 (39.57)	95.30 (35.62)	.54
6th month	62.77 (19.01)	69.84 (21.41)	.22
9th month	69.10 (26.70)	71.55 (32)	.79
HDL-C^d^ (mg/dL), mean (SD)			
Baseline	39.57 (7.38)	47.19 (13.86)	.02
6th month	42.91 (9.46)	44.94 (10)	.46
9th month	41.10 (8.09)	51.28 (21.92)	.05
Triglycerides (mg/dL), mean (SD)			
Baseline	184.26 (207.04)	146.11 (65.90)	.30
6th month	133.91 (53.19)	154.44 (119.35)	.45
9th month	142.85 (68.24)	133.88 (74.88)	.07
HbA_1c_^e^ (%), mean (SD)			
Baseline	6.13 (1.09)	6.02 (0.99)	.07
6th month	6.05 (0.80)	6 (0.70)	.83
9th month	6.12 (0.90)	6.07 (0.61)	.85
6-min walk distance (m), mean (SD)			
Baseline	433.35 (92.92)	414.46 (144.77)	.54
6th month	481.95 (80.26)	500.18 (93.70)	.48
9th month	510.56 (66.86)	508.87 (105.41)	.95

a Independent *t* test between groups.

b N/A: not applicable.

c LDL-C: low-density lipoprotein cholesterol.

d HDL-C: high-density lipoprotein cholesterol.

e HbA_1c_: hemoglobin A_1c_.

### Process Outcomes: App Acceptability and User Satisfaction

**Figure 3.**
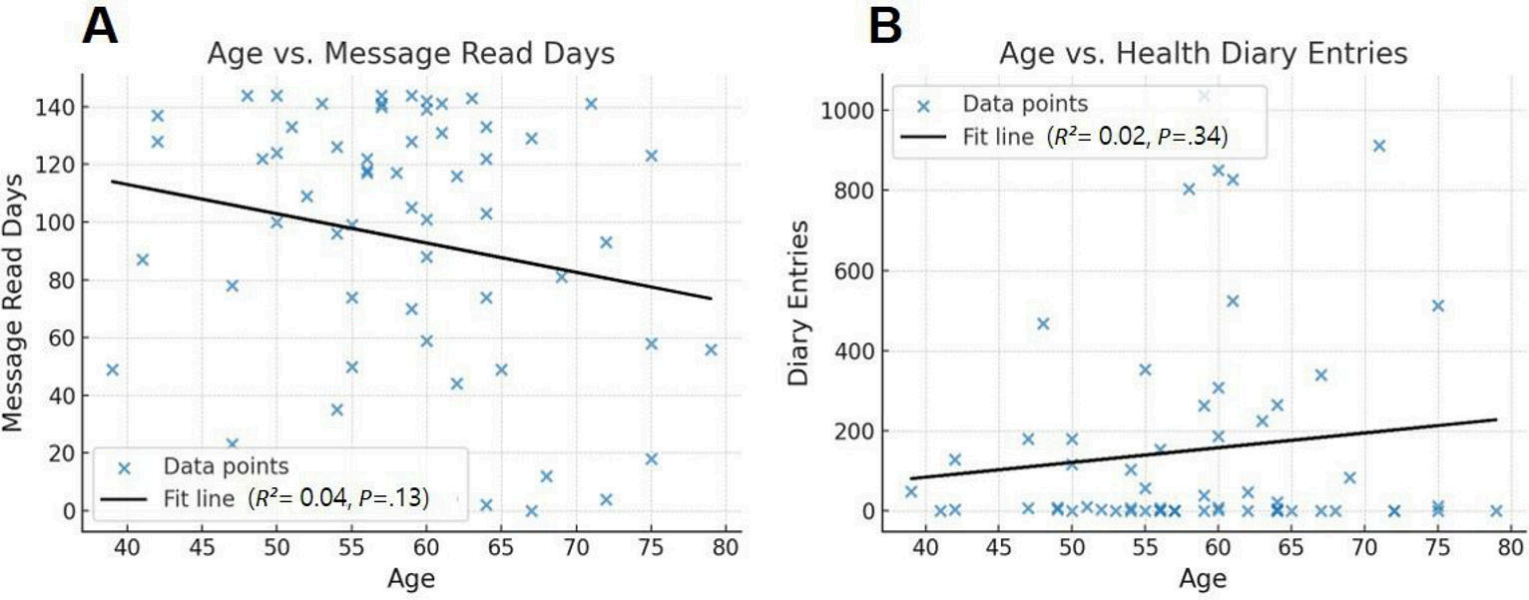
Correlation between age and AnSim app usage. (A) A scatter plot depicting the relationship between participant age and the number of days messages were read. (B) A scatter plot showing the relationship between participant age and the number of health diary entries recorded. Each point represents one participant, and the solid line represents the fitted linear regression.

Among 53 intervention participants who completed the satisfaction survey at 6 months, overall acceptability of the messaging intervention was high. Most respondents reported that the messages were easy to understand and helpful (n=45, 84.9%), expressed willingness to continue receiving messages (n=42, 79.2%), and indicated that they would recommend the app to others (84.9%). Detailed item-level acceptability and usability ratings are provided in Table S5 in Multimedia Appendix 1. We further explored whether engagement varied by participant characteristics. Linear regression analyses showed weak, nonsignificant associations between age and engagement metrics: age was not significantly associated with message-reading behavior (*R*²=0.04; *P*=.13; Figure 3A) or with diary engagement (*R*²=0.02; *P*=.34; Figure 3B). However, message reading frequency differed by education level, with elementary school graduates reading significantly fewer messages (*P*=.045; Table S6 in Multimedia Appendix 1). Satisfaction and health diary input frequency did not significantly differ by education level.

## Discussion

### Principal Findings and Interpretation

In this single-blinded randomized controlled trial, we evaluated the effectiveness of a patient-specific, smartphone-based messaging app (AnSim) on CV risk factor control in patients following PCI. Relative to our primary objectives, there were no significant between-group differences in changes in SBP or DBP. Within the intervention arm, greater engagement with messaging was associated with a higher likelihood of achieving guideline-recommended secondary prevention targets. Participants who experienced clinically relevant reductions in blood pressure at 9 months read more messages and demonstrated greater health behavior engagement, suggesting a potential dose–response relationship between digital engagement and risk factor control.

Several factors may have contributed to the lack of between-group differences in blood pressure. The participants had relatively well-controlled baseline blood pressure and lipid profiles, likely due to recent coronary intervention and optimal medical therapy. These ceiling effects may have limited the observable impact of the intervention. Moreover, adherence to medication and behavior change tend to be highest immediately following PCI, narrowing the margin for improvement and attenuating between-group differences [29]. A staged or adaptive approach, with reinforcement delivered later in recovery or targeted to patients with suboptimal baseline control, may be more likely to demonstrate incremental effects.

Several digital health interventions have demonstrated beneficial effects on CV outcomes, medication adherence, and behavior change [30,31]. Delivery of a comprehensive, app-supported rehabilitation program in the SMART-REHAB (SMARTphone-based early cardiac REHABilitation) trial was associated with improvements in physical recovery outcomes and greater enrollment and adherence to CR [32]. Compared to SMART-REHAB, which used structured exercise and real-time tracking, our AnSim intervention relied on passive, message-based behavioral prompting. This design distinction may explain differences in observed outcomes. Additionally, the baseline blood pressure of our participants was relatively well controlled due to recent PCI and optimal medical therapy, similar to the findings in SMART-REHAB where prescription rates for secondary prevention pharmacotherapy were consistently high. Accordingly, any incremental benefit of a low-intensity, message-only intervention on blood pressure is likely to be modest and may require longer follow-up or a higher-risk subgroup to be detectable.

Our results are consistent with the TEXT-ME study [18], which found that messaging helped maintain favorable risk profiles over time rather than lowering already well-controlled parameters. Additionally, our findings highlight the heterogeneity in responsiveness to digital interventions. Consistent with prior trials [33,34], our results demonstrate that engagement, not mere exposure, is a primary driver of digital intervention efficacy. Participants in the top half of message readership demonstrated greater health diary input and a higher likelihood of meeting at least 4 CV risk factor targets. Similarly, those who experienced improvements in blood pressure had significantly higher message exposure. This engagement-dependent effect parallels findings from a secondary analysis of a large CV text messaging trial, in which participants who actively engaged with the messages demonstrated significantly higher 12-month medication adherence than nonresponders, despite overall neutral trial results [35]. Such results support the view that the “dose” of digital engagement matters, and simply offering mobile health solutions may be insufficient without ensuring user uptake and sustained participation. Practically, it is essential to sustain participation through tailored content and timing, and to add a feedback loop that converts passive messages into actionable tasks (self-monitoring, goal tracking, and reinforcement). For users showing early drop-off, a stepped-care approach, such as automated reminders followed by brief coaching, may help restore engagement [36].

### Acceptability, Usability, and Durability of Effects

This trial represents an early real-world evaluation of AnSim, as acceptability and usability are central to interpreting scalability. Overall acceptability of the messaging content was high (eg, clarity/helpfulness, willingness to continue, and recommendation intent), supporting feasibility; however, overall usability ratings were comparatively lower, suggesting the presence of workflow friction that may limit sustained engagement [37]. To ensure digital equity, we implemented a simplified, user-friendly interface with large fonts and intuitive navigation, and provided face-to-face training at enrollment to overcome initial technical barriers. These measures likely contributed to our finding that age was not a significant determinant of message engagement. In contrast, education level remained an influencing factor; participants with lower education levels tended to read fewer messages, highlighting a persistent gap. This suggests that while simplified interfaces can bridge the age gap, addressing literacy barriers requires further tailoring [38]. Future interventions should focus on minimizing workflow friction and incorporating visual content with adaptive education levels to improve inclusivity and reduce disparities.

We observed a sustained and increased effect of the intervention over follow-up, which may reflect delayed behavior adoption or reinforcement over time. As shown in Table 4, the proportion of participants in the intervention group achieving multiple guideline-recommended risk factor targets was sustained or even slightly improved after the cessation of the 6-month messaging intervention. In contrast, the control group showed a marked decline in target achievement over the same period. These patterns are hypothesis-generating and suggest that the behavioral improvements facilitated by the AnSim messaging intervention may persist beyond the active intervention phase, supporting the hypothesis of a delayed or sustained effect. Given that secondary prevention requires lifelong adherence, interventions that promote sustained behavior change and long-term engagement may be particularly valuable, warranting longer-term evaluation.

### Strengths and Limitations

We used a randomized design with blinded outcome assessment and standardized follow-up to evaluate the incremental effect of a messaging intervention on risk-factor control after PCI. Strengths include randomized design, theory-based messaging content, integration of behavior change staging, and concurrent evaluation of clinical outcomes, acceptability, and intervention engagement. To support participant blinding, the Heart Keeper app was installed in both groups; Heart Keeper functioned as a simple diary-style tool for recording clinical information and did not deliver structured behavior-change messaging or coaching.

However, there are several limitations. First, the modest sample size and short follow-up period limited the power to detect differences in long-term CV outcomes. Second, we did not systematically capture detailed reasons for nonenrollment (including inability to use a smartphone), which limits the assessment of representativeness and generalizability, particularly for populations with lower digital access or literacy. Third, providing a diary-style logging application to both groups may have encouraged self-monitoring and reduced between-group separation. Fourth, our intervention lacked multimedia interactivity, real-time feedback, or structured exercise modules, which may have reduced efficacy. Regulatory restrictions in Korea also limited bidirectional communication, a component emphasized in SMART-REHAB as essential for behavior reinforcement. Furthermore, because Heart Keeper does not generate message-exposure metrics comparable to AnSim, objective engagement data were not available for the control group, precluding direct between-group comparisons of digital engagement intensity. Finally, we did not conduct qualitative interviews among participants with low engagement; future studies should incorporate user-centered qualitative methods to identify modifiable barriers and optimize equitable uptake.

### Implications for Practice and Future Research

The current study underscores the importance of personalization and engagement in digital secondary prevention support. While app-based interventions can extend the reach of CR programs, their impact depends heavily on user activation. Future versions of AnSim should incorporate co-design with patients and clinicians, formative usability testing, and stakeholder engagement to optimize fit-for-purpose delivery. Adaptive intervention timing (reinforcement initiated later in recovery or triggered by loss of control/engagement) may better align with patients’ evolving needs. Integrating app data with electronic medical records and care teams could facilitate more proactive medical adjustments, similar to nurse-led programs or digital case management models [39].

Larger trials with longer follow-up are needed to determine whether these interventions improve clinical outcomes such as hospital readmissions and mortality. Comparative effectiveness studies between full-scale digital rehabilitation programs and lighter interventions like AnSim will also help clarify the optimal design and intensity of mobile CR. Although messaging interventions may be low-cost per participant, implementation requires development, maintenance, and monitoring resources; future work should assess cost-effectiveness, including staff workload if escalation or bidirectional communication is introduced, and evaluate scalability in routine post-PCI care.

### Conclusions

This randomized trial demonstrated that a personalized, smartphone-based messaging intervention did not significantly improve blood pressure or lipid levels after PCI compared with usual care. In exploratory post hoc analyses within the intervention group, greater engagement with the AnSim app was associated with better risk-factor target attainment, with signals suggesting a larger potential benefit among participants with higher baseline blood pressure. These findings should be interpreted as hypothesis-generating and underscore engagement as a key determinant of potential impact for low-intensity mobile health strategies. Unlike comprehensive digital CR programs that incorporate structured exercise modules, real-time monitoring, or bidirectional coaching, AnSim was intentionally designed as a lightweight, message-mediated intervention that can be deployed with minimal resource burden. As a practical implication, these results suggest that real-world impact will likely depend less on message availability and more on implementation strategies that improve uptake and sustained engagement (eg, adaptive timing, stepped support, and integration into routine follow-up workflows). Future studies should test interventions designed to improve uptake and sustained participation, evaluate targeted or adaptive delivery for patients at higher residual risk, ensure digital equity for individuals with lower digital literacy, and assess longer-term clinical outcomes.

## Supplementary material

10.2196/81524 Multimedia Appendix 1 Comparison of the 2 apps, examples of developed messages, comparison of clinical events, guideline-recommended goals according to blood pressure improvement, acceptability and usability of the AnSim app at 6 months, and app utilization analysis according to education level.

10.2196/81524 Checklist 1 CONSORT-EHEALTH checklist (version 1.6.1).

10.2196/81524 Checklist 2 CONSORT 2025 checklist.

## Abbreviations

BP: blood pressure
CAD: coronary artery disease
CONSORT: Consolidated Standards of Reporting Trials
CONSORT-EHEALTH: Consolidated Standards of Reporting Trials of Electronic and Mobile Health Applications and Online Telehealth
CR: cardiac rehabilitation
CRIS: Clinical Research Information Service
CV: cardiovascular
DBP: diastolic blood pressure
HbA_1c_: hemoglobin A_1c_
LDL-C: low-density lipoprotein cholesterol
MCAR: missing completely at random
PCI: percutaneous coronary intervention
SBP: systolic blood pressure
SMART-REHAB: SMARTphone-based early cardiac REHABilitation
TEXT-ME: Tobacco, Exercise and Diet Messages
