# Supplementary material for: Personalized Smartphone Messaging for Secondary Prevention After Percutaneous Coronary Intervention: Randomized Controlled Trial
Source: J Med Internet Res. 2026 Apr 23;28:e81524. doi: 10.2196/81524 (PMC13105232; doi:10.2196/81524)
Supplement: Multimedia Appendix 1 [file jmir-v28-e81524-s001.docx]

**Supplemental Table 1. Comparison of two applications used in the study**

|  | | **AnSim** | **Heart Keeper** |
| --- | --- | --- | --- |
| **Producer** | | AnSim Investigators | The Korean Society of Cardiology |
| **Platform** | | Android,  (https://play.google.com/store/apps/details?id=kr.co.hanmihealthcare.ansim)  iOS (Not released publicly) | Android, (https://play.google.com/store/apps/details?id=com.m2comm.heart_p&hl=ko)  iOS (https://apps.apple.com/app/id1043400608) |
| **Language** | | Korean | Korean |
| **Contents** | **Messaging** | Messages encouraging & motivating healthy lifestyle & providing information (Text, Images, Movie clips) | None |
|  | **Self-tracking of biometric data** | Available | Available |
|  | **Information** | Patient-specific information via messaging | Encyclopedia format |
|  | **Feedback messages** | Yes | No |
| **Cost** | | Free | Free |

**Supplemental Table 2. Examples of developed messages**

| **Category** | **Example (English translation. The original messages were in Korean)** |
| --- | --- |
| General cardiovascular health and medications | Anti-platelet agents such as aspirin are very important for patients who received percutaneous coronary intervention. You should not arbitrarily stop the medicine for a tooth extraction or endoscopic examination. Please consult your doctor. |
| Nutrition | Eating too much salt can burden the heart, causing swelling and increased blood pressure. **(***Low-salt diet recipes Link***)** |
| Physical activity | Even only 30 minutes of exercise a day can reduce cardiac mortality and increase life expectancy. But do not overdo the exercise at the beginning. Increase your exercise time gradually. |
| Destress | Do you know that depression and coronary artery disease are correlated with each other? Coronary artery disease may lead to depression, and depression can increase the risk of coronary artery disease. |
| Smoking cessation | Free smoking cessation counseling. Free personalized 1:1 counseling at home or at work. (Phone number of national anti-smoking organization) |

**Supplemental Table 3. Comparison of clinical events**

|  | **Intervention**  **(N=60)** | **Control**  **(N=60)** | **Total**  **(N=120)** | ***P value** |
| --- | --- | --- | --- | --- |
| All clinical events | 0 (0%) | 2 (3.3%) | 2 (1.7%) | 0.496 |
| MACE | 0 (0%) | 1 (1.7%) | 1 (0.8%) | 1.000 |
| All-cause death | 0 (0%) | 0 (0%) | 0 (0%) | n/a |
| Myocardial infarction | 0 (0%) | 0 (0%) | 0 (0%) | n/a |
| Coronary revascularization | 0 (0%) | 1 (1.7%) | 1 (0.8%) | 1.000 |
| Readmission | 0 (0%) | 2 (3.3%) | 2 (1.7%) | 0.496 |

*Independent t-test between Control and Intervention groups.

**Supplemental Table 4. Guideline-recommended goals according to BP improvement**

|  | |  | BP  improved group | BP  not-improved group | Relative risk (95% CI) | *P value |
| --- | --- | --- | --- | --- | --- | --- |
|  | | Baseline  6^th^ month  9^th^ month | N= 23  N=23  N=21 | N=37  N=35  N=33 |  |  |
| LDL <70mg/dL | |  |  |  |  |  |
|  | Baseline |  | 6 (26.1%) | 12 (32.4%) | 0.804 (0.351-1.845) | 0.773 |
|  | 6^th^ month |  | 16 (69.6%) | 15 (42.9%) | 1.623 (1.016-2.593) | 0.062 |
|  | 9^th^ month |  | 13 (61.9%) | 11 (33.3%) | 1.857 (1.032-3.343) | 0.052 |
| BP <140/90 | |  |  |  |  |  |
|  | Baseline |  | 16 (69.6%) | 35 (94.6%) | 0.735 (0.555-0.974) | 0.021 |
|  | 6^th^ month |  | 17 (73.9%) | 22 (62.9%) | 1.176 (0.827-1.672) | 0.410 |
|  | 9^th^ month |  | 15 (71.4%) | 14 (42.4%) | 1.684 (1.041-2.723) | 0.052 |
| Regular exercise | |  |  |  |  |  |
|  | Baseline |  | 9 (31.1%) | 17 (45.9%) | 0.852 (0.459-1.580) | 0.789 |
|  | 6^th^ month |  | 18 (78.3%) | 23 (65.7%) | 1.191 (0.863-1.643) | 0.384 |
|  | 9^th^ month |  | 17 (81.0%) | 19 (57.6%) | 1.406 (0.982-2.013) | 0.138 |
| Non-smoker | |  |  |  |  |  |
|  | Baseline |  | 16 (69.6%) | 27 (73.0%) | 0.953 (0.683-1.331) | 0.777 |
|  | 6^th^ month |  | 19 (82.6%) | 24 (68.6%) | 1.205 (0.899-1.614) | 0.359 |
|  | 9^th^ month |  | 18 (85.7%) | 24 (72.7%) | 1.179 (0.898-1.547) | 0.329 |
| BMI <25, kg/m^2^ | |  |  |  |  |  |
|  | Baseline |  | 6 (26.1%) | 16 (43.2%) | 0.603 (0.276-1.317) | 0.271 |
|  | 6^th^ month |  | 7 (30.4%) | 15 (42.9%) | 0.710 (0.343-1.469) | 0.413 |
|  | 9^th^ month |  | 5 (23.8%) | 11 (33.3%) | 0.714 (0.289-1.765) | 0.549 |
| Four of five | |  |  |  |  |  |
|  | Baseline |  | 4 (17.4%) | 13 (35.1%) | 0.495 (0.183-1.335) | 0.157 |
|  | 6^th^ month |  | 13 (56.5%) | 10 (28.6%) | 1.978 (1.049-3.732) | 0.054 |
|  | 9^th^ month |  | 11 (52.4%) | 8 (24.2%) | 2.161 (1.043-4.475) | 0.045 |
| Five of five | |  |  |  |  |  |
|  | Baseline |  | 0 (0.0%) | 0 (0.0%) | n/a | n/a |
|  | 6^th^ month |  | 4 (17.4%) | 1 (2.9%) | 6.087 (0.725-51.081) | 0.075 |
|  | 9^th^ month |  | 4 (19.0%) | 2 (6.1%) | 3.143 (0.630-15.673) | 0.193 |
| At least four of five | |  |  |  |  |  |
|  | Baseline |  | 4 (17.4%) | 13 (35.1%) | 0.497 (0.172–1.438) | 0.174 |
|  | 6^th^ month |  | 17 (73.9%) | 11 (31.4%) | 2.170 (1.057–4.457) | 0.041 |
|  | 9^th^ month |  | 15 (71.4%) | 10 (30.3%) | 2.270 (1.046–4.927) | 0.045 |

*Independent t-test between BP improved and BP not-improved groups.

**Supplemental Table 5. Acceptability and usability of the AnSim app at 6 months**

| **Question** | **Total N** | **1 Not at all** | **2 Not so** | **3 Average** | **4 Yes** | **5 Very much** | **4+5 Positive** |
| --- | --- | --- | --- | --- | --- | --- | --- |
| Overall, I was satisfied with using the AnSim app. | 53 | 0 (0.0%) | 2 (3.8%) | 11 (20.8%) | 28 (52.8%) | 12 (22.6%) | 40 (75.5%) |
| I would recommend the AnSim app to others. | 53 | 0 (0.0%) | 1 (1.9%) | 7 (13.2%) | 31 (58.5%) | 14 (26.4%) | 45 (84.9%) |
| I intend to continue using the AnSim app. | 53 | 0 (0.0%) | 4 (7.5%) | 7 (13.2%) | 30 (56.6%) | 12 (22.6%) | 42 (79.2%) |
| [Overall layout] The screens and contents were appropriately designed and well presented. | 53 | 0 (0.0%) | 5 (9.4%) | 10 (18.9%) | 27 (50.9%) | 11 (20.8%) | 38 (71.7%) |
| [Readability] The message font size, access to logs/records, and illustrative materials were appropriate and easy to understand. | 53 | 0 (0.0%) | 3 (5.7%) | 5 (9.4%) | 35 (66.0%) | 10 (18.9%) | 45 (84.9%) |
| [Functionality] Patient note entry, alarm settings, and navigation between menus were convenient and well organized. | 52 | 0 (0.0%) | 3 (5.8%) | 11 (21.2%) | 29 (55.8%) | 9 (17.3%) | 38 (73.1%) |
| [Usability] Receiving messages, receiving/recording information, and checking rules were easy to do in the app. | 53 | 2 (3.8%) | 8 (15.1%) | 16 (30.2%) | 19 (35.8%) | 8 (15.1%) | 27 (50.9%) |

*Self-reported questionnaire administered at the 6-month follow-up. Responses were recorded on a 5-point Likert scale (1=not at all, 2=not so, 3=average, 4=yes, 5=very much). Data are presented as n (%). Positive response defined as 4 or 5.

**Supplemental Table 6. App utilization analysis according to education level**

| **Education level** |  | |  | |
| --- | --- | --- | --- | --- |
|  | **Number** | **Number of days the messaged was read** | **Number of health diary inputs** | **Satisfaction with AnSim application** |
| Elementary School | 7 | 54.7±42.9 | 55.3±50.6 | 3.5±0.84 |
| Middle School | 3 | 127.0±26.9 | 276.0±276.0 | 3.6±0.60 |
| High School | 29 | 98.6±44.0 | 195.65±58.2 | 3.7±0.60 |
| University | 20 | 101.6±36.0 | 138.2±57.6 | 3.6±0.55 |
| Graduate School | 1 | n/a | n/a | n/a |
|  |  | P=0.045 | P=0.651 | P=0.997 |

Data are presented as mean ± standard deviation.
