# Supplementary material for: Personalized Smartphone Messaging for Secondary Prevention After Percutaneous Coronary Intervention: Randomized Controlled Trial
Source: J Med Internet Res. 2026 Apr 23;28:e81524. doi: 10.2196/81524 (PMC13105232; doi:10.2196/81524)
Supplement: Checklist 1 [file jmir-v28-e81524-s002.pdf]

CONSORT-EHEALTH (V 1.6.1) - Submission/Publication Form

The CONSORT-EHEALTH checklist is intended for authors of randomized trials evaluating web-based and Internet-based applications/interventions, including mobile interventions, electronic games (incl multiplayer games), social media, certain telehealth applications, and other interactive and/or networked electronic applications. Some of the items (e.g. all subitems under item 5 - description of the intervention) may also be applicable for other study designs.

The goal of the CONSORT EHEALTH checklist and guideline is to be
a) a guide for reporting for authors of RCTs,
b) to form a basis for appraisal of an ehealth trial (in terms of validity)

CONSORT-EHEALTH items/subitems are MANDATORY reporting items for studies published in the Journal of Medical Internet Research and other journals / scientific societies endorsing the checklist.

Items numbered 1., 2., 3., 4a., 4b etc are original CONSORT or CONSORT-NPT (non-pharmacologic treatment) items.
Items with Roman numerals (i., ii, iii, iv etc.) are CONSORT-EHEALTH extensions/clarifications.

As the CONSORT-EHEALTH checklist is still considered in a formative stage, we would ask that you also RATE ON A SCALE OF 1-5 how important/useful you feel each item is FOR THE PURPOSE OF THE CHECKLIST and reporting guideline (optional).

Mandatory reporting items are marked with a red *.
In the textboxes, either copy & paste the relevant sections from your manuscript into this form - please include any quotes from your manuscript in QUOTATION MARKS,
or answer directly by providing additional information not in the manuscript, or elaborating on why the item was not relevant for this study.

YOUR ANSWERS WILL BE PUBLISHED AS A SUPPLEMENTARY FILE TO YOUR PUBLICATION IN JMIR AND ARE CONSIDERED PART OF YOUR PUBLICATION (IF ACCEPTED).
Please fill in these questions diligently. Information will not be copyedited, so please use proper spelling and grammar, use correct capitalization, and avoid abbreviations.

DO NOT FORGET TO SAVE AS PDF _AND_ CLICK THE SUBMIT BUTTON SO YOUR ANSWERS ARE IN OUR DATABASE !!!

Citation Suggestion (if you append the pdf as Appendix we suggest to cite this paper in the caption):
Eysenbach G, CONSORT-EHEALTH Group
CONSORT-EHEALTH: Improving and Standardizing Evaluation Reports of Web-based and Mobile Health Interventions
J Med Internet Res 2011;13(4):e126

URL: http://www.jmir.org/2011/4/e126/
doi: 10.2196/jmir.1923
PMID: 22209829

galiard4@gmail.com 계정 전환

초안이 저장되지 않음

비공개

* 표시는 필수 질문임

Your name *

First Last

Eung Ju Kim

Primary Affiliation (short), City, Country *

University of Toronto, Toronto, Canada

Korea University Guro Hospital, Seoul, Korea

Your e-mail address *

abc@gmail.com

withnoel@empas.com

Title of your manuscript *

Provide the (draft) title of your manuscript.

Personalized Smartphone Messaging to Support Cardiac Rehabilitation After PCI: A Randomized Controlled Trial

Name of your App/Software/Intervention *

If there is a short and a long/alternate name, write the short name first and add the long name in brackets.

AnSim (Application for Self-improvement)

Evaluated Version (if any)

e.g. "V1", "Release 2017-03-01", "Version 2.0.27913"

내 답변

Language(s) *

What language is the intervention/app in? If multiple languages are available, separate by comma (e.g. "English, French")

Korean

URL of your Intervention Website or App

e.g. a direct link to the mobile app on app in appstore (itunes, Google Play), or URL of the website. If the intervention is a DVD or hardware, you can also link to an Amazon page.

내 답변

URL of an image/screenshot (optional)

내 답변

Accessibility *

Can an enduser access the intervention presently?

- ◯ access is free and open
- ◯ access only for special usergroups, not open
- ◯ access is open to everyone, but requires payment/subscription/in-app purchases
- ◉ app/intervention no longer accessible
- ◯ 기타:

Primary Medical Indication/Disease/Condition *

e.g. "Stress", "Diabetes", or define the target group in brackets after the condition, e.g. "Autism (Parents of children with)", "Alzheimers (Informal Caregivers of)"

who had undergone PCI recently (high CV risk

Primary Outcomes measured in trial *

comma-separated list of primary outcomes reported in the trial

The primary outcome was change in blood pre

Secondary/other outcomes

Are there any other outcomes the intervention is expected to affect?

Secondary outcomes were lipid profiles (total cholesterol, LDL-C, HDL-C, triglycerides), HbA1c, 6-minute walk distance, self-reported smoking status, and venous carboxyhemoglobin (CO-Hb) levels as a biochemical indicator of smoking

Recommended "Dose" *

What do the instructions for users say on how often the app should be used?

- (•) Approximately Daily
- ( ) Approximately Weekly
- ( ) Approximately Monthly
- ( ) Approximately Yearly
- ( ) "as needed"
- ( ) 기타:

Approx. Percentage of Users (starters) still using the app as recommended after 3 months *

- ( ) unknown / not evaluated
- ( ) 0-10%
- ( ) 11-20%
- ( ) 21-30%
- ( ) 31-40%
- ( ) 41-50%
- ( ) 51-60%
- ( ) 61-70%
- ( ) 71%-80%
- ( ) 81-90%
- (•) 91-100%
- ( ) 기타:

Overall, was the app/intervention effective? *

- ( ) yes: all primary outcomes were significantly better in intervention group vs control
- ( ) partly: SOME primary outcomes were significantly better in intervention group vs control
- (•) no statistically significant difference between control and intervention
- ( ) potentially harmful: control was significantly better than intervention in one or more outcomes
- ( ) inconclusive: more research is needed
- ( ) 기타:

Article Preparation Status/Stage *

At which stage in your article preparation are you currently (at the time you fill in this form)

- ( ) not submitted yet - in early draft status
- ( ) not submitted yet - in late draft status, just before submission
- (•) submitted to a journal but not reviewed yet
- ( ) submitted to a journal and after receiving initial reviewer comments
- ( ) submitted to a journal and accepted, but not published yet
- ( ) published
- ( ) 기타:

Journal *

If you already know where you will submit this paper (or if it is already submitted), please provide the journal name (if it is not JMIR, provide the journal name under "other")

- ○ not submitted yet / unclear where I will submit this
- ◉ Journal of Medical Internet Research (JMIR)
- ○ JMIR mHealth and UHealth
- ○ JMIR Serious Games
- ○ JMIR Mental Health
- ○ JMIR Public Health
- ○ JMIR Formative Research
- ○ Other JMIR sister journal
- ○ 기타:

Is this a full powered effectiveness trial or a pilot/feasibility trial? *

- ◉ Pilot/feasibility
- ○ Fully powered

Manuscript tracking number *

If this is a JMIR submission, please provide the manuscript tracking number under "other" (The ms tracking number can be found in the submission acknowledgement email, or when you login as author in JMIR. If the paper is already published in JMIR, then the ms tracking number is the four-digit number at the end of the DOI, to be found at the bottom of each published article in JMIR)

- ○ no ms number (yet) / not (yet) submitted to / published in JMIR
- ◉ 기타: 81524

TITLE AND ABSTRACT

1a) TITLE: Identification as a randomized trial in the title

1a) Does your paper address CONSORT item 1a? *

I.e does the title contain the phrase "Randomized Controlled Trial"? (if not, explain the reason under "other")

- (●) yes
- ( ) 기타:

1a-i) Identify the mode of delivery in the title

Identify the mode of delivery. Preferably use “web-based” and/or “mobile” and/or “electronic game” in the title. Avoid ambiguous terms like “online”, “virtual”, “interactive”. Use “Internet-based” only if Intervention includes non-web-based Internet components (e.g. email), use “computer-based” or “electronic” only if offline products are used. Use “virtual” only in the context of “virtual reality” (3-D worlds). Use “online” only in the context of “online support groups”. Complement or substitute product names with broader terms for the class of products (such as “mobile” or “smart phone” instead of “iphone”), especially if the application runs on different platforms.

	1	2	3	4	5	
subitem not at all important	( )	( )	( )	( )	(●)	essential

선택해제

Does your paper address subitem 1a-i? *

Copy and paste relevant sections from manuscript title (include quotes in quotation marks "like this" to indicate direct quotes from your manuscript), or elaborate on this item by providing additional information not in the ms, or briefly explain why the item is not applicable/relevant for your study

Personalized "Smartphone Messaging" to Support Cardiac Rehabilitation After PCI: A Randomized Controlled Trial

1a-ii) Non-web-based components or important co-interventions in title

Mention non-web-based components or important co-interventions in title, if any (e.g., “with telephone support”).

	1	2	3	4	5	
subitem not at all important	○	○	○	○	◉	essential

선택해제

Does your paper address subitem 1a-ii?

Copy and paste relevant sections from manuscript title (include quotes in quotation marks "like this" to indicate direct quotes from your manuscript), or elaborate on this item by providing additional information not in the ms, or briefly explain why the item is not applicable/relevant for your study

"Personalized" Smartphone Messaging to Support Cardiac Rehabilitation After PCI: A Randomized Controlled Trial

1a-iii) Primary condition or target group in the title

Mention primary condition or target group in the title, if any (e.g., “for children with Type I Diabetes”) Example: A Web-based and Mobile Intervention with Telephone Support for Children with Type I Diabetes: Randomized Controlled Trial

	1	2	3	4	5	
subitem not at all important	○	○	○	○	◉	essential

선택해제

Does your paper address subitem 1a-iii? *

Copy and paste relevant sections from manuscript title (include quotes in quotation marks "like this" to indicate direct quotes from your manuscript), or elaborate on this item by providing additional information not in the ms, or briefly explain why the item is not applicable/relevant for your study

Personalized Smartphone Messaging to Support Cardiac Rehabilitation "After PCI": A Randomized Controlled Trial

1b) ABSTRACT: Structured summary of trial design, methods, results, and conclusions

NPT extension: Description of experimental treatment, comparator, care providers, centers, and blinding status.

1b-i) Key features/functionalities/components of the intervention and comparator in the METHODS section of the ABSTRACT

Mention key features/functionalities/components of the intervention and comparator in the abstract. If possible, also mention theories and principles used for designing the site. Keep in mind the needs of systematic reviewers and indexers by including important synonyms. (Note: Only report in the abstract what the main paper is reporting. If this information is missing from the main body of text, consider adding it)

	1	2	3	4	5	
subitem not at all important	○	○	○	○	◉	essential

선택해제

Does your paper address subitem 1b-i? *

Copy and paste relevant sections from the manuscript abstract (include quotes in quotation marks "like this" to indicate direct quotes from your manuscript), or elaborate on this item by providing additional information not in the ms, or briefly explain why the item is not applicable/relevant for your study

"randomly assigned (1:1) to receive either a smartphone-based message intervention via the AnSim app plus usual care (intervention group) or usual care alone (control group)."

1b-ii) Level of human involvement in the METHODS section of the ABSTRACT

Clarify the level of human involvement in the abstract, e.g., use phrases like “fully automated” vs. “therapist/nurse/care provider/physician-assisted” (mention number and expertise of providers involved, if any). (Note: Only report in the abstract what the main paper is reporting. If this information is missing from the main body of text, consider adding it)

	1	2	3	4	5	
subitem not at all important	○	○	○	●	○	essential

선택해제

Does your paper address subitem 1b-ii?

Copy and paste relevant sections from the manuscript abstract (include quotes in quotation marks "like this" to indicate direct quotes from your manuscript), or elaborate on this item by providing additional information not in the ms, or briefly explain why the item is not applicable/relevant for your study

"The intervention comprised personalized educational and motivational messages tailored by behavioral stage and risk profile, delivered 6 times per week for 6 months."

1b-iii) Open vs. closed, web-based (self-assessment) vs. face-to-face assessments in the METHODS section of the ABSTRACT

Mention how participants were recruited (online vs. offline), e.g., from an open access website or from a clinic or a closed online user group (closed usergroup trial), and clarify if this was a purely web-based trial, or there were face-to-face components (as part of the intervention or for assessment). Clearly say if outcomes were self-assessed through questionnaires (as common in web-based trials). Note: In traditional offline trials, an open trial (open-label trial) is a type of clinical trial in which both the researchers and participants know which treatment is being administered. To avoid confusion, use “blinded” or “unblinded” to indicated the level of blinding instead of “open”, as “open” in web-based trials usually refers to “open access” (i.e. participants can self-enrol). (Note: Only report in the abstract what the main paper is reporting. If this information is missing from the main body of text, consider adding it)

	1	2	3	4	5	
subitem not at all important	○	○	◉	○	○	essential

선택해제

Does your paper address subitem 1b-iii?

Copy and paste relevant sections from the manuscript abstract (include quotes in quotation marks "like this" to indicate direct quotes from your manuscript), or elaborate on this item by providing additional information not in the ms, or briefly explain why the item is not applicable/relevant for your study

“This was a single-blinded, 2-arm randomized controlled trial conducted at 2 hospitals in Korea.”

1b-iv) RESULTS section in abstract must contain use data

Report number of participants enrolled/assessed in each group, the use/uptake of the intervention (e.g., attrition/adherence metrics, use over time, number of logins etc.), in addition to primary/secondary outcomes. (Note: Only report in the abstract what the main paper is reporting. If this information is missing from the main body of text, consider adding it)

	1	2	3	4	5	
subitem not at all important	○	○	○	◉	○	essential

선택해제

Does your paper address subitem 1b-iv?

Copy and paste relevant sections from the manuscript abstract (include quotes in quotation marks "like this" to indicate direct quotes from your manuscript), or elaborate on this item by providing additional information not in the ms, or briefly explain why the item is not applicable/relevant for your study

"A total of 120 patients who underwent PCI within the prior month was randomly assigned (1:1) to receive either a smartphone-based message intervention via the AnSim app plus usual care (intervention group) or usual care alone (control group)."

"Patients with improved blood pressure at 9 months had read significantly more messages (110.3 vs 83.3 days, P = .02)."

"Overall user satisfaction with the app was high, with 87% finding messages helpful and 81% expressing a desire to continue receiving them."

1b-v) CONCLUSIONS/DISCUSSION in abstract for negative trials

Conclusions/Discussions in abstract for negative trials: Discuss the primary outcome - if the trial is negative (primary outcome not changed), and the intervention was not used, discuss whether negative results are attributable to lack of uptake and discuss reasons. (Note: Only report in the abstract what the main paper is reporting. If this information is missing from the main body of text, consider adding it)

	1	2	3	4	5	
subitem not at all important	○	○	○	○	◉	essential

선택해제

Does your paper address subitem 1b-v?

Copy and paste relevant sections from the manuscript abstract (include quotes in quotation marks "like this" to indicate direct quotes from your manuscript), or elaborate on this item by providing additional information not in the ms, or briefly explain why the item is not applicable/relevant for your study

“There were no significant differences between groups in blood pressure or secondary endpoints at 6 or 9 months.”

“However, within the intervention group, patients who read more messages (>median) showed greater health diary engagement and were more likely to meet ≥4 recommended CV risk factor targets at 9 months (69.0% vs. 19.2%, P <0.001).”

“This suggests that individualized, theory-based mHealth interventions may be beneficial for motivated users and warrant further long-term study.”

INTRODUCTION

2a) In INTRODUCTION: Scientific background and explanation of rationale

2a-i) Problem and the type of system/solution

Describe the problem and the type of system/solution that is object of the study: intended as stand-alone intervention vs. incorporated in broader health care program? Intended for a particular patient population? Goals of the intervention, e.g., being more cost-effective to other interventions, replace or complement other solutions? (Note: Details about the intervention are provided in “Methods” under 5)

	1	2	3	4	5	
subitem not at all important	○	○	○	○	◉	essential

선택해제

Does your paper address subitem 2a-i? *

Copy and paste relevant sections from the manuscript (include quotes in quotation marks "like this" to indicate direct quotes from your manuscript), or elaborate on this item by providing additional information not in the ms, or briefly explain why the item is not applicable/relevant for your study

“Cardiac rehabilitation (CR) improves outcomes after percutaneous coronary intervention (PCI), but participation remains suboptimal due to barriers such as distance, cost, and lack of referral.”

“Recent advances in mobile communication technologies have introduced new opportunities to overcome these barriers.”

“In this context, we developed a smartphone-based, patient-specific messaging application called AnSim and conducted a randomized controlled trial to evaluate its clinical efficacy and user acceptability.”

2a-ii) Scientific background, rationale: What is known about the (type of) system

Scientific background, rationale: What is known about the (type of) system that is the object of the study (be sure to discuss the use of similar systems for other conditions/diagnoses, if appropiate), motivation for the study, i.e. what are the reasons for and what is the context for this specific study, from which stakeholder viewpoint is the study performed, potential impact of findings [2]. Briefly justify the choice of the comparator.

	1	2	3	4	5	
subitem not at all important	○	○	○	○	◉	essential

선택해제

Does your paper address subitem 2a-ii? *

Copy and paste relevant sections from the manuscript (include quotes in quotation marks "like this" to indicate direct quotes from your manuscript), or elaborate on this item by providing additional information not in the ms, or briefly explain why the item is not applicable/relevant for your study

"Recent advances in mobile communication technologies have introduced new opportunities to overcome these barriers. The widespread availability of mobile phones and smartphones enables rapid, low-cost, individualized health messaging based on user characteristics such as age, sex, and location."

"In the context of CR, SMS-based interventions have demonstrated effectiveness in improving medication adherence and maintaining blood pressure control in CAD patients (14, 15). As smartphone capabilities have expanded, mobile applications can now deliver multimedia messages, personalized content, and interactive features."

"Prior studies have begun to explore the use of app-based CR interventions with promising feasibility and patient satisfaction (16). However, the clinical impact of such tools on long-term CV risk factor control remains uncertain."

"In this context, we developed a smartphone-based, patient-specific messaging application called AnSim and conducted a randomized controlled trial to evaluate its clinical efficacy and user acceptability."

2b) In INTRODUCTION: Specific objectives or hypotheses

Does your paper address CONSORT subitem 2b? *

Copy and paste relevant sections from the manuscript (include quotes in quotation marks "like this" to indicate direct quotes from your manuscript), or elaborate on this item by providing additional information not in the ms, or briefly explain why the item is not applicable/relevant for your study

“This study aimed to assess whether personalized messaging—tailored according to behavioral stage, comorbidities, and user preferences—could enhance CV risk factor control and support CR in patients with CAD following percutaneous coronary intervention (PCI).”

METHODS

3a) Description of trial design (such as parallel, factorial) including allocation ratio

Does your paper address CONSORT subitem 3a? *

Copy and paste relevant sections from the manuscript (include quotes in quotation marks "like this" to indicate direct quotes from your manuscript), or elaborate on this item by providing additional information not in the ms, or briefly explain why the item is not applicable/relevant for your study

“The clinical trial was designed as a single-blinded, 2-arm randomized controlled trial with a total follow-up period of 9 months—comprising 6 months of intervention and 3 months of additional observation (Figure 1).”

“A total of 120 patients who had undergone PCI within 1 month was enrolled and randomly assigned in a 1:1 ratio to the intervention group (AnSim application + usual care) or the control group (usual care only).”

3b) Important changes to methods after trial commencement (such as eligibility criteria), with reasons

Does your paper address CONSORT subitem 3b? *

Copy and paste relevant sections from the manuscript (include quotes in quotation marks "like this" to indicate direct quotes from your manuscript), or elaborate on this item by providing additional information not in the ms, or briefly explain why the item is not applicable/relevant for your study

“The clinical trial was designed as a single-blinded, 2-arm randomized controlled trial with a total follow-up period of 9 months—comprising 6 months of intervention and 3 months of additional observation.”

“A total of 120 patients who had undergone PCI within 1 month was enrolled and randomly assigned in a 1:1 ratio to the intervention group (AnSim application + usual care) or the control group (usual care only).”

3b-i) Bug fixes, Downtimes, Content Changes

Bug fixes, Downtimes, Content Changes: ehealth systems are often dynamic systems. A description of changes to methods therefore also includes important changes made on the intervention or comparator during the trial (e.g., major bug fixes or changes in the functionality or content) (5-iii) and other “unexpected events” that may have influenced study design such as staff changes, system failures/downtimes, etc. [2].

	1	2	3	4	5	
subitem not at all important	○	◉	○	○	○	essential

선택해제

Does your paper address subitem 3b-i?

Copy and paste relevant sections from the manuscript (include quotes in quotation marks "like this" to indicate direct quotes from your manuscript), or elaborate on this item by providing additional information not in the ms, or briefly explain why the item is not applicable/relevant for your study

If needed, we will add following sentence in the manuscript.

“No changes in app functionality, message content, or technical downtimes occurred during the intervention period.”

4a) Eligibility criteria for participants

Does your paper address CONSORT subitem 4a? *

Copy and paste relevant sections from the manuscript (include quotes in quotation marks "like this" to indicate direct quotes from your manuscript), or elaborate on this item by providing additional information not in the ms, or briefly explain why the item is not applicable/relevant for your study

"Eligible participants were adults (≥18 years) who had undergone PCI within the previous 1 month and were able to use a smartphone. Patients with cognitive dysfunction (e.g., stroke or dementia) or significant psychiatric disorders (e.g., anxiety, depression) were excluded. PCI was performed in patients with significant anatomic stenosis (>50% in the left main or >70% in non-left main coronary arteries) or physiologic ischemia (fractional flow reserve <0.80) and included cases of ST-elevation myocardial infarction (STEMI), non-ST elevation acute coronary syndrome (NSTE-ACS), unstable angina, and stable angina. All patients received guideline-directed medical therapy."

4a-i) Computer / Internet literacy

Computer / Internet literacy is often an implicit “de facto” eligibility criterion - this should be explicitly clarified.

	1	2	3	4	5	
subitem not at all important	○	○	○	○	◉	essential

선택해제

Does your paper address subitem 4a-i?

Copy and paste relevant sections from the manuscript (include quotes in quotation marks "like this" to indicate direct quotes from your manuscript), or elaborate on this item by providing additional information not in the ms, or briefly explain why the item is not applicable/relevant for your study

“Eligible participants were adults (≥18 years) who had undergone PCI within the previous 1 month and were able to use a smartphone.”

“Patients with cognitive dysfunction (e.g., stroke or dementia) or significant psychiatric disorders (e.g., anxiety, depression) were excluded.”

“Exclusion criteria included inability to use a smartphone, non-fluency in Korean, prior coronary artery bypass graft surgery, uncontrolled arrhythmia or heart failure, severe chronic obstructive pulmonary disease or asthma, end-stage renal disease requiring dialysis, or terminal malignancy.”

4a-ii) Open vs. closed, web-based vs. face-to-face assessments:

Open vs. closed, web-based vs. face-to-face assessments: Mention how participants were recruited (online vs. offline), e.g., from an open access website or from a clinic, and clarify if this was a purely web-based trial, or there were face-to-face components (as part of the intervention or for assessment), i.e., to what degree got the study team to know the participant. In online-only trials, clarify if participants were quasi-anonymous and whether having multiple identities was possible or whether technical or logistical measures (e.g., cookies, email confirmation, phone calls) were used to detect/prevent these.

	1	2	3	4	5	
subitem not at all important	○	○	○	○	◉	essential

선택해제

Does your paper address subitem 4a-ii? *

Copy and paste relevant sections from the manuscript (include quotes in quotation marks "like this" to indicate direct quotes from your manuscript), or elaborate on this item by providing additional information not in the ms, or briefly explain why the item is not applicable/relevant for your study

“The clinical trial was designed as a single-blinded, 2-arm randomized controlled trial... The trial was conducted at 2 sites in Korea: a secondary general hospital (Sejong General Hospital) and a tertiary academic hospital (Korea University Guro Hospital).”

“A total of 120 patients who had undergone PCI within 1 month was enrolled...”

“All participants provided written informed consent.”

4a-iii) Information giving during recruitment

Information given during recruitment. Specify how participants were briefed for recruitment and in the informed consent procedures (e.g., publish the informed consent documentation as appendix, see also item X26), as this information may have an effect on user self-selection, user expectation and may also bias results.

	1	2	3	4	5	
subitem not at all important	○	○	○	◉	○	essential

선택해제

Does your paper address subitem 4a-iii?

Copy and paste relevant sections from the manuscript (include quotes in quotation marks "like this" to indicate direct quotes from your manuscript), or elaborate on this item by providing additional information not in the ms, or briefly explain why the item is not applicable/relevant for your study

“The trial was registered with the Clinical Research Information Service (CRIS; KCT0002361) and approved by the Institutional Review Board of Korea University Guro Hospital (IRB No. MD16037-002). All participants provided written informed consent.”

4b) Settings and locations where the data were collected

Does your paper address CONSORT subitem 4b? *

Copy and paste relevant sections from the manuscript (include quotes in quotation marks "like this" to indicate direct quotes from your manuscript), or elaborate on this item by providing additional information not in the ms, or briefly explain why the item is not applicable/relevant for your study

“The trial was conducted at 2 sites in Korea: a secondary general hospital (Sejong General Hospital) and a tertiary academic hospital (Korea University Guro Hospital).”

4b-i) Report if outcomes were (self-)assessed through online questionnaires

Clearly report if outcomes were (self-)assessed through online questionnaires (as common in web-based trials) or otherwise.

	1	2	3	4	5	
subitem not at all important	○	○	○	○	●	essential

선택해제

Does your paper address subitem 4b-i? *

Copy and paste relevant sections from the manuscript (include quotes in quotation marks "like this" to indicate direct quotes from your manuscript), or elaborate on this item by providing additional information not in the ms, or briefly explain why the item is not applicable/relevant for your study

“Participants were assessed at baseline, 6 months, and 9 months. The primary outcome was change in blood pressure, measured after 10 minutes of seated rest using a standardized automatic device.”

“Secondary outcomes were lipid profiles (total cholesterol, LDL-C, HDL-C, triglycerides), HbA1c, 6-minute walk distance, self-reported smoking status, and venous carboxyhemoglobin (CO-Hb) levels as a biochemical indicator of smoking.”

“Process evaluation included app usage metrics… and patient-reported satisfaction with and utility of the messages, measured via a post-intervention questionnaire.”

4b-ii) Report how institutional affiliations are displayed

Report how institutional affiliations are displayed to potential participants [on ehealth media], as affiliations with prestigious hospitals or universities may affect volunteer rates, use, and reactions with regards to an intervention.(Not a required item – describe only if this may bias results)

	1	2	3	4	5	
subitem not at all important	○	◉	○	○	○	essential

선택해제

Does your paper address subitem 4b-ii?

Copy and paste relevant sections from the manuscript (include quotes in quotation marks "like this" to indicate direct quotes from your manuscript), or elaborate on this item by providing additional information not in the ms, or briefly explain why the item is not applicable/relevant for your study

Participants were recruited in person at two hospitals (Korea University Guro Hospital and Sejong General Hospital), not through an online platform. The AnSim app was not publicly advertised or available for open self-enrollment, and no institutional branding was presented via open web interfaces. Therefore, participants were informed of institutional affiliations during standard in-person consent procedures, not via eHealth media or public-facing digital materials.

5) The interventions for each group with sufficient details to allow replication, including how and when they were actually administered

5-i) Mention names, credential, affiliations of the developers, sponsors, and owners

Mention names, credential, affiliations of the developers, sponsors, and owners [6] (if authors/evaluators are owners or developer of the software, this needs to be declared in a “Conflict of interest” section or mentioned elsewhere in the manuscript).

	1	2	3	4	5	
subitem not at all important	○	○	○	○	◉	essential

선택해제

Does your paper address subitem 5-i?

Copy and paste relevant sections from the manuscript (include quotes in quotation marks "like this" to indicate direct quotes from your manuscript), or elaborate on this item by providing additional information not in the ms, or briefly explain why the item is not applicable/relevant for your study

“We gratefully acknowledge the contributions of Yeonghun Song and Gyeongho Jung from the IT business division at Hanmi Healthcare for their work in application development and study operations.”

“This research was supported by a grant from the Korea Health Industry Development Institute (KHIDI), funded by the Ministry of Health and Welfare, Republic of Korea (grant number: HI16C0483). The funders had no role in the design of the study, data collection and analysis, interpretation of data, or writing of the manuscript.”

5-ii) Describe the history/development process

Describe the history/development process of the application and previous formative evaluations (e.g., focus groups, usability testing), as these will have an impact on adoption/use rates and help with interpreting results.

	1	2	3	4	5	
subitem not at all important	○	○	○	○	◉	essential

선택해제

Does your paper address subitem 5-ii?

Copy and paste relevant sections from the manuscript (include quotes in quotation marks "like this" to indicate direct quotes from your manuscript), or elaborate on this item by providing additional information not in the ms, or briefly explain why the item is not applicable/relevant for your study

We have published design paper already in JMIR Medical Informatics. (JMIR Med Inform 2021;9(12):e23285) doi: 10.2196/23285

5-iii) Revisions and updating

Revisions and updating. Clearly mention the date and/or version number of the application/intervention (and comparator, if applicable) evaluated, or describe whether the intervention underwent major changes during the evaluation process, or whether the development and/or content was "frozen" during the trial. Describe dynamic components such as news feeds or changing content which may have an impact on the replicability of the intervention (for unexpected events see item 3b).

	1	2	3	4	5	
subitem not at all important	○	○	◉	○	○	essential

선택해제

Does your paper address subitem 5-iii?

Copy and paste relevant sections from the manuscript (include quotes in quotation marks "like this" to indicate direct quotes from your manuscript), or elaborate on this item by providing additional information not in the ms, or briefly explain why the item is not applicable/relevant for your study

The message content was fixed after development and did not dynamically update (i.e., “messages were never repeated for the same individual”).

Messages were tailored and scheduled, but not changed or added during the intervention phase.

5-iv) Quality assurance methods

Provide information on quality assurance methods to ensure accuracy and quality of information provided [1], if applicable.

	1	2	3	4	5	
subitem not at all important	○	○	○	○	◉	essential

선택해제

Does your paper address subitem 5-iv?

Copy and paste relevant sections from the manuscript (include quotes in quotation marks "like this" to indicate direct quotes from your manuscript), or elaborate on this item by providing additional information not in the ms, or briefly explain why the item is not applicable/relevant for your study

“Each message contained 40–140 Korean characters and was adapted from international guidelines and official educational resources from CV health-related academic societies.”

“A total of 450 messages (90 per category) was initially developed and reviewed for clinical accuracy, literacy level, and relevance.”

“Subsequently, 200 potential users evaluated each message using a 5-point Likert scale for readability and usefulness.”

“Messages were refined accordingly…”

5-v) Ensure replicability by publishing the source code, and/or providing screenshots/screen-capture video, and/or providing flowcharts of the algorithms used

Ensure replicability by publishing the source code, and/or providing screenshots/screen-capture video, and/or providing flowcharts of the algorithms used. Replicability (i.e., other researchers should in principle be able to replicate the study) is a hallmark of scientific reporting.

	1	2	3	4	5	
subitem not at all important	○	○	○	◉	○	essential

선택해제

Does your paper address subitem 5-v?

Copy and paste relevant sections from the manuscript (include quotes in quotation marks "like this" to indicate direct quotes from your manuscript), or elaborate on this item by providing additional information not in the ms, or briefly explain why the item is not applicable/relevant for your study

“The messaging content was informed by 26 established behavior change techniques derived from models such as the information-motivation-behavioral skills model, theory of planned behavior, social cognitive theory, and operant conditioning.”

“To match message content to individual readiness for change, we applied a simplified transtheoretical model with 3 behavioral stages… Each patient’s behavioral stage for each message category was reassessed monthly through the app, and messaging was adapted accordingly.”

“Messages were drawn from the 5 categories… with 5 standard messages per week plus 1 additional message targeting an individual’s weakest behavior domain.”

5-vi) Digital preservation

Digital preservation: Provide the URL of the application, but as the intervention is likely to change or disappear over the course of the years; also make sure the intervention is archived (Internet Archive, webcitation.org, and/or publishing the source code or screenshots/videos alongside the article). As pages behind login screens cannot be archived, consider creating demo pages which are accessible without login.

	1	2	3	4	5	
subitem not at all important	○	○	◉	○	○	essential

선택해제

Does your paper address subitem 5-vi?

Copy and paste relevant sections from the manuscript (include quotes in quotation marks "like this" to indicate direct quotes from your manuscript), or elaborate on this item by providing additional information not in the ms, or briefly explain why the item is not applicable/relevant for your study

To ensure digital preservation of the intervention, a demo document including screenshots, version history, and app registration details of the AnSim application has been archived at Zenodo: [10.5281/zenodo.16672624].

5-vii) Access

Access: Describe how participants accessed the application, in what setting/context, if they had to pay (or were paid) or not, whether they had to be a member of specific group. If known, describe how participants obtained “access to the platform and Internet” [1]. To ensure access for editors/reviewers/readers, consider to provide a “backdoor” login account or demo mode for reviewers/readers to explore the application (also important for archiving purposes, see vi).

	1	2	3	4	5	
subitem not at all important	○	○	○	◉	○	essential

선택해제

Does your paper address subitem 5-vii? *

Copy and paste relevant sections from the manuscript (include quotes in quotation marks "like this" to indicate direct quotes from your manuscript), or elaborate on this item by providing additional information not in the ms, or briefly explain why the item is not applicable/relevant for your study

“A total of 120 patients who had undergone PCI within 1 month was enrolled and randomly assigned in a 1:1 ratio to the intervention group (AnSim application + usual care) or the control group (usual care only).”

“All participants were provided with a general health monitoring app (‘Heart Keeper’)... Only those in the intervention group received the AnSim application in addition to Heart Keeper.”

“Research nurses trained in allocation concealment and participant instruction managed all app installations.”

5-viii) Mode of delivery, features/functionalities/components of the intervention and comparator, and the theoretical framework

Describe mode of delivery, features/functionalities/components of the intervention and comparator, and the theoretical framework [6] used to design them (instructional strategy [1], behaviour change techniques, persuasive features, etc., see e.g., [7, 8] for terminology). This includes an in-depth description of the content (including where it is coming from and who developed it) [1],” whether [and how] it is tailored to individual circumstances and allows users to track their progress and receive feedback” [6]. This also includes a description of communication delivery channels and – if computer-mediated communication is a component – whether communication was synchronous or asynchronous [6]. It also includes information on presentation strategies [1], including page design principles, average amount of text on pages, presence of hyperlinks to other resources, etc. [1].

	1	2	3	4	5	
subitem not at all important	○	○	○	○	◉	essential

선택해제

Does your paper address subitem 5-viii? *

Copy and paste relevant sections from the manuscript (include quotes in quotation marks "like this" to indicate direct quotes from your manuscript), or elaborate on this item by providing additional information not in the ms, or briefly explain why the item is not applicable/relevant for your study

1. Mode of Delivery:
From the Abstract and Methods – Delivery of Messaging Intervention:

“Participants in the intervention group received 6 messages per week over a 24-week period. Messages were sent randomly among chosen times (9:00 AM, 12:00 PM, or 3:00 PM) Monday through Saturday.”

“The content was personalized based on baseline characteristics (e.g., presence of diabetes, smoking status) and behavioral stage.”

“Messages were drawn from the 5 categories mentioned above, with 5 standard messages per week plus 1 additional message targeting an individual’s weakest behavior domain.”

→ Mode: Asynchronous, push-based message delivery through a smartphone app (AnSim).

2. Features / Functionalities / Components:
From the Methods – Message Development:

“Health experts drafted messages in 5 thematic categories: 1) general CV health and medications, 2) nutrition, 3) physical activity, 4) stress management, and 5) smoking cessation.”

“The AnSim and Heart Keeper apps allowed optional tracking of health metrics (e.g., blood pressure, glucose, exercise, diet).”

“Participants… received weekly feedback messages based on their logged health data, sent by a designated healthcare provider.”

→ Key components included:

Tailored educational messages

Health diary/self-tracking tools

Weekly personalized feedback

User-specific content adaptation based on behavioral stage and comorbidity

3. Theoretical Framework:
“The messaging content was informed by 26 established behavior change techniques derived from models such as the information-motivation-behavioral skills model, theory of planned behavior, social cognitive theory, and operant conditioning.”

“To match message content to individual readiness for change, we applied a simplified transtheoretical model with 3 behavioral stages: (1) pre-contemplation, (2) contemplation/preparation, and (3) action/maintenance.”

→ Theoretical foundation is clearly defined and appropriate for behavior change interventions.

5-ix) Describe use parameters

Describe use parameters (e.g., intended "doses" and optimal timing for use). Clarify what instructions or recommendations were given to the user, e.g., regarding timing, frequency, heaviness of use, if any, or was the intervention used ad libitum.

	1	2	3	4	5	
subitem not at all important	○	○	○	○	◉	essential

선택해제

Does your paper address subitem 5-ix?

Copy and paste relevant sections from the manuscript (include quotes in quotation marks "like this" to indicate direct quotes from your manuscript), or elaborate on this item by providing additional information not in the ms, or briefly explain why the item is not applicable/relevant for your study

- Delivery of Messaging Intervention section:
"Participants in the intervention group received 6 messages per week over a 24-week period."

"Messages were sent randomly among chosen times (9:00 AM, 12:00 PM, or 3:00 PM) Monday through Saturday."

"Each patient's behavioral stage for each message category was reassessed monthly through the app, and messaging was adapted accordingly."

"To enhance engagement, participants in the intervention group also received weekly feedback messages based on their logged health data, sent by a designated healthcare provider."

"All participants received brief onboarding at enrollment."

5-x) Clarify the level of human involvement

Clarify the level of human involvement (care providers or health professionals, also technical assistance) in the e-intervention or as co-intervention (detail number and expertise of professionals involved, if any, as well as “type of assistance offered, the timing and frequency of the support, how it is initiated, and the medium by which the assistance is delivered”. It may be necessary to distinguish between the level of human involvement required for the trial, and the level of human involvement required for a routine application outside of a RCT setting (discuss under item 21 – generalizability).

	1	2	3	4	5	
subitem not at all important	○	○	○	◉	○	essential

선택해제

Does your paper address subitem 5-x?

Copy and paste relevant sections from the manuscript (include quotes in quotation marks "like this" to indicate direct quotes from your manuscript), or elaborate on this item by providing additional information not in the ms, or briefly explain why the item is not applicable/relevant for your study

- From the Methods – Delivery of Messaging Intervention:

“All participants received brief onboarding at enrollment.”

“To enhance engagement, participants in the intervention group also received weekly feedback messages based on their logged health data, sent by a designated healthcare provider.”

- From the Methods – Blinding and Randomization:

“Research nurses trained in allocation concealment and participant instruction managed all app installations.”

5-xi) Report any prompts/reminders used

Report any prompts/reminders used: Clarify if there were prompts (letters, emails, phone calls, SMS) to use the application, what triggered them, frequency etc. It may be necessary to distinguish between the level of prompts/reminders required for the trial, and the level of prompts/reminders for a routine application outside of a RCT setting (discuss under item 21 – generalizability).

	1	2	3	4	5	
subitem not at all important	○	○	○	◉	○	essential

선택해제

Does your paper address subitem 5-xi? *

Copy and paste relevant sections from the manuscript (include quotes in quotation marks "like this" to indicate direct quotes from your manuscript), or elaborate on this item by providing additional information not in the ms, or briefly explain why the item is not applicable/relevant for your study

“Participants in the intervention group received 6 messages per week over a 24-week period. Messages were sent randomly among chosen times (9:00 AM, 12:00 PM, or 3:00 PM) Monday through Saturday.”

“To enhance engagement, participants in the intervention group also received weekly feedback messages based on their logged health data.”

“Messages were never repeated for the same individual.”

5-xii) Describe any co-interventions (incl. training/support)

Describe any co-interventions (incl. training/support): Clearly state any interventions that are provided in addition to the targeted eHealth intervention, as ehealth intervention may not be designed as stand-alone intervention. This includes training sessions and support [1]. It may be necessary to distinguish between the level of training required for the trial, and the level of training for a routine application outside of a RCT setting (discuss under item 21 – generalizability.

	1	2	3	4	5	
subitem not at all important	○	○	○	●	○	essential

선택해제

Does your paper address subitem 5-xii? *

Copy and paste relevant sections from the manuscript (include quotes in quotation marks "like this" to indicate direct quotes from your manuscript), or elaborate on this item by providing additional information not in the ms, or briefly explain why the item is not applicable/relevant for your study

“All participants received brief onboarding at enrollment.”

“To minimize performance and measurement bias, all participants were provided with a general health monitoring app (‘Heart Keeper’)... Only those in the intervention group received the AnSim application in addition to Heart Keeper.”

“Research nurses trained in allocation concealment and participant instruction managed all app installations.”

6a) Completely defined pre-specified primary and secondary outcome measures, including how and when they were assessed

Does your paper address CONSORT subitem 6a? *

Copy and paste relevant sections from the manuscript (include quotes in quotation marks "like this" to indicate direct quotes from your manuscript), or elaborate on this item by providing additional information not in the ms, or briefly explain why the item is not applicable/relevant for your study

“The primary outcome was change in blood pressure at 6 and 9 months.”

“Participants were assessed at baseline, 6 months, and 9 months.”

“Blood pressure was measured after 10 minutes of seated rest using a standardized automatic device (HEM-7080IT, OMRON Healthcare, Japan).”

“Secondary outcomes were lipid profiles (total cholesterol, LDL-C, HDL-C, triglycerides), HbA1c, 6-minute walk distance, self-reported smoking status, and venous carboxyhemoglobin (CO-Hb) levels as a biochemical indicator of smoking.”

“Process evaluation included app usage metrics… and patient-reported satisfaction with and utility of the messages, measured via a post-intervention questionnaire.”

6a-i) Online questionnaires: describe if they were validated for online use and apply CHERRIES items to describe how the questionnaires were designed/deployed

If outcomes were obtained through online questionnaires, describe if they were validated for online use and apply CHERRIES items to describe how the questionnaires were designed/deployed [9].

	1	2	3	4	5	
subitem not at all important	○	○	○	●	○	essential

선택해제

Does your paper address subitem 6a-i?

Copy and paste relevant sections from manuscript text

“Process evaluation included app usage metrics… and patient-reported satisfaction with and utility of the messages, measured via a post-intervention questionnaire.”

“Self-reported smoking status… was assessed alongside CO-Hb levels.”

6a-ii) Describe whether and how “use” (including intensity of use/dosage) was defined/measured/monitored

Describe whether and how “use” (including intensity of use/dosage) was defined/measured/monitored (logins, logfile analysis, etc.). Use/adoption metrics are important process outcomes that should be reported in any ehealth trial.

	1	2	3	4	5	
subitem not at all important	○	○	○	○	◉	essential

선택해제

Does your paper address subitem 6a-ii?

Copy and paste relevant sections from manuscript text

"Process evaluation included app usage metrics (e.g., message delivery success, message reading frequency, health data input frequency) and patient-reported satisfaction with and utility of the messages, measured via a post-intervention questionnaire."

"Among intervention group participants, the frequency of message reading was analyzed as a marker of engagement. Patients were stratified into high (upper 50%) and low (lower 50%) message readers. High readers accessed more than twice as many messages on average (130.5 vs. 50.6 messages, P<.001) and had significantly greater engagement in health diary input (250.2 vs. 46.8 entries, P=.001)."

6a-iii) Describe whether, how, and when qualitative feedback from participants was obtained

Describe whether, how, and when qualitative feedback from participants was obtained (e.g., through emails, feedback forms, interviews, focus groups).

	1	2	3	4	5	
subitem not at all important	○	○	○	◉	○	essential

선택해제

Does your paper address subitem 6a-iii?

Copy and paste relevant sections from manuscript text

“Process evaluation included app usage metrics… and patient-reported satisfaction with and utility of the messages, measured via a post-intervention questionnaire.”

6b) Any changes to trial outcomes after the trial commenced, with reasons

Does your paper address CONSORT subitem 6b? *

Copy and paste relevant sections from the manuscript (include quotes in quotation marks "like this" to indicate direct quotes from your manuscript), or elaborate on this item by providing additional information not in the ms, or briefly explain why the item is not applicable/relevant for your study

No changes were made to the primary or secondary outcome measures after trial commencement.

7a) How sample size was determined

NPT: When applicable, details of whether and how the clustering by care provides or centers was addressed

7a-i) Describe whether and how expected attrition was taken into account when calculating the sample size

Describe whether and how expected attrition was taken into account when calculating the sample size.

	1	2	3	4	5	
subitem not at all important	○	○	○	○	●	essential

선택해제

Does your paper address subitem 7a-i?

Copy and paste relevant sections from manuscript title (include quotes in quotation marks "like this" to indicate direct quotes from your manuscript), or elaborate on this item by providing additional information not in the ms, or briefly explain why the item is not applicable/relevant for your study

After reviewing, I found that the following content was missing, so I will add to the manuscript.

"In the Text-Me study similar to this study (JAMA. 2015; 314(12): 1255-1263), in six months, blood pressure increased by 6%, resulting in about 8 mmHg difference between the blood pressures between the two groups. Based on these results, the minimum sample size calculated using Medcalc (Ver.16.2.1, Medcalc Software, Mariakerke, Belgium) is 121. [α = 0.05, β = 0.20 (Power = 0.8)]. In this study, 60 patients were assigned to each group, and a total of 120 patients participated."

7b) When applicable, explanation of any interim analyses and stopping guidelines

Does your paper address CONSORT subitem 7b? *

Copy and paste relevant sections from the manuscript (include quotes in quotation marks "like this" to indicate direct quotes from your manuscript), or elaborate on this item by providing additional information not in the ms, or briefly explain why the item is not applicable/relevant for your study

No interim analyses or stopping guidelines were planned or conducted, as the study was a parallel-group RCT with a fixed 9-month follow-up period and minimal expected risk.

8a) Method used to generate the random allocation sequence

NPT: When applicable, how care providers were allocated to each trial group

Does your paper address CONSORT subitem 8a? *

Copy and paste relevant sections from the manuscript (include quotes in quotation marks "like this" to indicate direct quotes from your manuscript), or elaborate on this item by providing additional information not in the ms, or briefly explain why the item is not applicable/relevant for your study

"Participants were randomly assigned to either the intervention or control group using a centralized, computer-generated randomization table with a 1:1 allocation ratio and block size of 4."

8b) Type of randomisation; details of any restriction (such as blocking and block size)

Does your paper address CONSORT subitem 8b? *

Copy and paste relevant sections from the manuscript (include quotes in quotation marks "like this" to indicate direct quotes from your manuscript), or elaborate on this item by providing additional information not in the ms, or briefly explain why the item is not applicable/relevant for your study

"Participants were randomly assigned to either the intervention or control group using a centralized, computer-generated randomization table with a 1:1 allocation ratio and block size of 4."

9) Mechanism used to implement the random allocation sequence (such as sequentially numbered containers), describing any steps taken to conceal the sequence until interventions were assigned

Does your paper address CONSORT subitem 9? *

Copy and paste relevant sections from the manuscript (include quotes in quotation marks "like this" to indicate direct quotes from your manuscript), or elaborate on this item by providing additional information not in the ms, or briefly explain why the item is not applicable/relevant for your study

"Participants were randomly assigned to either the intervention or control group using a centralized, computer-generated randomization table with a 1:1 allocation ratio and block size of 4. To minimize performance and measurement bias, all participants were provided with a general health monitoring app (“Heart Keeper”) developed by the Korean Society of Cardiology. Only those in the intervention group received the AnSim application in addition to Heart Keeper. Blinding was maintained by ensuring that outcome assessors and investigators were unaware of group allocation. Research nurses trained in allocation concealment and participant instruction managed all app installations."

10) Who generated the random allocation sequence, who enrolled participants, and who assigned participants to interventions

Does your paper address CONSORT subitem 10? *

Copy and paste relevant sections from the manuscript (include quotes in quotation marks "like this" to indicate direct quotes from your manuscript), or elaborate on this item by providing additional information not in the ms, or briefly explain why the item is not applicable/relevant for your study

"Research nurses trained in allocation concealment and participant instruction managed all app installations."

11a) If done, who was blinded after assignment to interventions (for example, participants, care providers, those assessing outcomes) and how

NPT: Whether or not administering co-interventions were blinded to group assignment

11a-i) Specify who was blinded, and who wasn’t

Specify who was blinded, and who wasn’t. Usually, in web-based trials it is not possible to blind the participants [1, 3] (this should be clearly acknowledged), but it may be possible to blind outcome assessors, those doing data analysis or those administering co-interventions (if any).

	1	2	3	4	5	
subitem not at all important	○	○	○	◉	○	essential

선택해제

Does your paper address subitem 11a-i? *

Copy and paste relevant sections from the manuscript (include quotes in quotation marks "like this" to indicate direct quotes from your manuscript), or elaborate on this item by providing additional information not in the ms, or briefly explain why the item is not applicable/relevant for your study

"Blinding was maintained by ensuring that outcome assessors and investigators were unaware of group allocation. Research nurses trained in allocation concealment and participant instruction managed all app installations. Participants were instructed not to disclose their app usage to study staff during follow-up visits." but if needed, we can add following sentences;

“Due to the nature of the intervention, which involved receiving personalized smartphone messages, participants were not blinded after group allocation.”

11a-ii) Discuss e.g., whether participants knew which intervention was the “intervention of interest” and which one was the “comparator”

Informed consent procedures (4a-ii) can create biases and certain expectations - discuss e.g., whether participants knew which intervention was the “intervention of interest” and which one was the “comparator”.

	1	2	3	4	5	
subitem not at all important	○	○	○	◉	○	essential

선택해제

Does your paper address subitem 11a-ii?

Copy and paste relevant sections from the manuscript (include quotes in quotation marks "like this" to indicate direct quotes from your manuscript), or elaborate on this item by providing additional information not in the ms, or briefly explain why the item is not applicable/relevant for your study

"To minimize performance and measurement bias, all participants were provided with a general health monitoring app (“Heart Keeper”) developed by the Korean Society of Cardiology."

11b) If relevant, description of the similarity of interventions

(this item is usually not relevant for ehealth trials as it refers to similarity of a placebo or sham intervention to a active medication/intervention)

Does your paper address CONSORT subitem 11b? *

Copy and paste relevant sections from the manuscript (include quotes in quotation marks "like this" to indicate direct quotes from your manuscript), or elaborate on this item by providing additional information not in the ms, or briefly explain why the item is not applicable/relevant for your study

"To minimize performance and measurement bias, all participants were provided with a general health monitoring app (“Heart Keeper”) developed by the Korean Society of Cardiology. Only those in the intervention group received the AnSim application in addition to Heart Keeper."

"A comparison between the 2 applications is listed in Supplemental Table 1."

12a) Statistical methods used to compare groups for primary and secondary outcomes

NPT: When applicable, details of whether and how the clustering by care providers or centers was addressed

Does your paper address CONSORT subitem 12a? *

Copy and paste relevant sections from the manuscript (include quotes in quotation marks "like this" to indicate direct quotes from your manuscript), or elaborate on this item by providing additional information not in the ms, or briefly explain why the item is not applicable/relevant for your study

"All analyses were performed on an intention-to-treat basis using SPSS version 20.0 (IBM Corp, Chicago, IL, USA). Continuous variables are presented as means with standard deviations; categorical variables are reported as counts and percentages. Normality was assessed using the Shapiro-Wilk test and variance homogeneity by F-test. Between-group comparisons for continuous variables were performed using independent-samples t-tests. Categorical variables were compared using Pearson's chi-square or Fisher's exact tests, as appropriate. To examine group-by-time interactions for repeated measures, 2-way mixed analysis of variance (ANOVA) was used. Relative risks and 95% confidence intervals for outcomes were estimated using Cox proportional-hazards models. All statistical tests were 2-sided, and P<.05 was considered statistically significant."

12a-i) Imputation techniques to deal with attrition / missing values

Imputation techniques to deal with attrition / missing values: Not all participants will use the intervention/comparator as intended and attrition is typically high in ehealth trials. Specify how participants who did not use the application or dropped out from the trial were treated in the statistical analysis (a complete case analysis is strongly discouraged, and simple imputation techniques such as LOCF may also be problematic [4]).

	1	2	3	4	5	
subitem not at all important	○	○	◉	○	○	essential

선택해제

Does your paper address subitem 12a-i? *

Copy and paste relevant sections from the manuscript (include quotes in quotation marks "like this" to indicate direct quotes from your manuscript), or elaborate on this item by providing additional information not in the ms, or briefly explain why the item is not applicable/relevant for your study

Participants with missing outcome data were not imputed. Sensitivity analyses were not conducted, which is a limitation.

12b) Methods for additional analyses, such as subgroup analyses and adjusted analyses

Does your paper address CONSORT subitem 12b? *

Copy and paste relevant sections from the manuscript (include quotes in quotation marks "like this" to indicate direct quotes from your manuscript), or elaborate on this item by providing additional information not in the ms, or briefly explain why the item is not applicable/relevant for your study

"Since the intervention using the AnSim application was message-mediated, the efficacy of the intervention could be dependent on participant message utilization. Among intervention group participants, the frequency of message reading was analyzed as a marker of engagement. Patients were stratified into high (upper 50%) and low (lower 50%) message readers (Table 4)."

"In a subgroup analysis, patients in the intervention group were categorized based on whether they demonstrated improvement in blood pressure by 9 months (n=23) or not (n=33). Responders had significantly higher baseline systolic and diastolic blood pressures but showed greater reductions over time. At 9 months, mean systolic blood pressure was lower in the responder group (121.4 vs. 130.8 mmHg, P=.024), and diastolic blood pressure was similarly reduced (75.9 vs. 84.3 mmHg, P=.030; Table 5)."

X26) REB/IRB Approval and Ethical Considerations [recommended as subheading under "Methods"] (not a CONSORT item)

X26-i) Comment on ethics committee approval

	1	2	3	4	5	
subitem not at all important	○	○	○	○	◉	essential

선택해제

Does your paper address subitem X26-i?

Copy and paste relevant sections from the manuscript (include quotes in quotation marks "like this" to indicate direct quotes from your manuscript), or elaborate on this item by providing additional information not in the ms, or briefly explain why the item is not applicable/relevant for your study

"The trial was registered with the Clinical Research Information Service (CRIS; KCT0002361) and approved by the Institutional Review Board of Korea University Guro Hospital (IRB No. MD16037-002). All participants provided written informed consent."

x26-ii) Outline informed consent procedures

Outline informed consent procedures e.g., if consent was obtained offline or online (how? Checkbox, etc.?), and what information was provided (see 4a-ii). See [6] for some items to be included in informed consent documents.

	1	2	3	4	5	
subitem not at all important	○	○	○	○	◉	essential

선택해제

Does your paper address subitem X26-ii?

Copy and paste relevant sections from the manuscript (include quotes in quotation marks "like this" to indicate direct quotes from your manuscript), or elaborate on this item by providing additional information not in the ms, or briefly explain why the item is not applicable/relevant for your study

“All participants provided written informed consent.”

X26-iii) Safety and security procedures

Safety and security procedures, incl. privacy considerations, and any steps taken to reduce the likelihood or detection of harm (e.g., education and training, availability of a hotline)

	1	2	3	4	5	
subitem not at all important	○	○	○	◉	○	essential

선택해제

Does your paper address subitem X26-iii?

Copy and paste relevant sections from the manuscript (include quotes in quotation marks "like this" to indicate direct quotes from your manuscript), or elaborate on this item by providing additional information not in the ms, or briefly explain why the item is not applicable/relevant for your study

Not explicitly addressed in the manuscript. We will add following sentences;

“Data privacy was ensured through encryption and secure servers, and participants were provided with contact details to report any concerns.”

RESULTS

13a) For each group, the numbers of participants who were randomly assigned, received intended treatment, and were analysed for the primary outcome

NPT: The number of care providers or centers performing the intervention in each group and the number of patients treated by each care provider in each center

Does your paper address CONSORT subitem 13a? *

Copy and paste relevant sections from the manuscript (include quotes in quotation marks "like this" to indicate direct quotes from your manuscript), or elaborate on this item by providing additional information not in the ms, or briefly explain why the item is not applicable/relevant for your study

"A total of 120 patients was enrolled between December 2016 and April 2018 and randomized into the intervention group (n=60) and the control group (n=60). Follow-up completion rates were high: 58 (96.7%) in the intervention group and 57 (95.0%) in the control group at 6 months and 54 (90.0%) and 56 (93.3%), respectively, at 9 months."

13b) For each group, losses and exclusions after randomisation, together with reasons

Does your paper address CONSORT subitem 13b? (NOTE: Preferably, this is shown in a CONSORT flow diagram) *

Copy and paste relevant sections from the manuscript (include quotes in quotation marks "like this" to indicate direct quotes from your manuscript), or elaborate on this item by providing additional information not in the ms, or briefly explain why the item is not applicable/relevant for your study

"Reasons for attrition included consent withdrawal (n=2 in each group) and loss to follow-up (n=4 in the intervention group, n=2 in the control group)."

13b-i) Attrition diagram

Strongly recommended: An attrition diagram (e.g., proportion of participants still logging in or using the intervention/comparator in each group plotted over time, similar to a survival curve) or other figures or tables demonstrating usage/dose/engagement.

	1	2	3	4	5	
subitem not at all important	○	○	○	◉	○	essential

선택해제

Does your paper address subitem 13b-i?

Copy and paste relevant sections from the manuscript or cite the figure number if applicable (include quotes in quotation marks "like this" to indicate direct quotes from your manuscript), or elaborate on this item by providing additional information not in the ms, or briefly explain why the item is not applicable/relevant for your study

The manuscript includes a participant flow diagram (Figure 1) showing overall participant across randomization, follow-up, and analysis. Additionally, Tables 2 describe the number of participants completing follow-up at 6 and 9 months. If necessary, I will include the number of Drop-out number and the reason in Figure 1.

14a) Dates defining the periods of recruitment and follow-up

Does your paper address CONSORT subitem 14a? *

Copy and paste relevant sections from the manuscript (include quotes in quotation marks "like this" to indicate direct quotes from your manuscript), or elaborate on this item by providing additional information not in the ms, or briefly explain why the item is not applicable/relevant for your study

"A total of 120 patients was enrolled between December 2016 and April 2018 and randomized into the intervention group (n=60) and the control group (n=60)."

14a-i) Indicate if critical “secular events” fell into the study period

Indicate if critical “secular events” fell into the study period, e.g., significant changes in Internet resources available or “changes in computer hardware or Internet delivery resources”

	1	2	3	4	5	
subitem not at all important	◉	○	○	○	○	essential

선택해제

Does your paper address subitem 14a-i?

Copy and paste relevant sections from the manuscript (include quotes in quotation marks "like this" to indicate direct quotes from your manuscript), or elaborate on this item by providing additional information not in the ms, or briefly explain why the item is not applicable/relevant for your study

No secular events are reported in the manuscript.

14b) Why the trial ended or was stopped (early)

Does your paper address CONSORT subitem 14b? *

Copy and paste relevant sections from the manuscript (include quotes in quotation marks "like this" to indicate direct quotes from your manuscript), or elaborate on this item by providing additional information not in the ms, or briefly explain why the item is not applicable/relevant for your study

This study was completed as planned and was not stopped early.

15) A table showing baseline demographic and clinical characteristics for each group

NPT: When applicable, a description of care providers (case volume, qualification, expertise, etc.) and centers (volume) in each group

Does your paper address CONSORT subitem 15? *

Copy and paste relevant sections from the manuscript (include quotes in quotation marks "like this" to indicate direct quotes from your manuscript), or elaborate on this item by providing additional information not in the ms, or briefly explain why the item is not applicable/relevant for your study

The baseline demographic and clinical characteristics for each group are clearly presented in the manuscript in:

“Table 1. Baseline characteristics of the study participants.”

15-i) Report demographics associated with digital divide issues

In ehealth trials it is particularly important to report demographics associated with digital divide issues, such as age, education, gender, social-economic status, computer/Internet/ehealth literacy of the participants, if known.

	1	2	3	4	5	
subitem not at all important	○	○	○	○	◉	essential

선택해제

Does your paper address subitem 15-i? *

Copy and paste relevant sections from the manuscript (include quotes in quotation marks "like this" to indicate direct quotes from your manuscript), or elaborate on this item by providing additional information not in the ms, or briefly explain why the item is not applicable/relevant for your study

"Baseline characteristics, including age, gender, educational status, clinical diagnosis, and medication history, were collected and are summarized in Table 1."

16) For each group, number of participants (denominator) included in each analysis and whether the analysis was by original assigned groups

16-i) Report multiple "denominators" and provide definitions

Report multiple "denominators" and provide definitions: Report N's (and effect sizes) "across a range of study participation [and use] thresholds" [1], e.g., N exposed, N consented, N used more than x times, N used more than y weeks, N participants "used" the intervention/comparator at specific pre-defined time points of interest (in absolute and relative numbers per group). Always clearly define "use" of the intervention.

	1	2	3	4	5	
subitem not at all important	○	○	○	○	◉	essential

선택해제

Does your paper address subitem 16-i? *

Copy and paste relevant sections from the manuscript (include quotes in quotation marks "like this" to indicate direct quotes from your manuscript), or elaborate on this item by providing additional information not in the ms, or briefly explain why the item is not applicable/relevant for your study

Relevant manuscript excerpts:

Total enrolled: “A total of 120 patients was enrolled between December 2016 and April 2018 and randomized into the intervention group (n=60) and the control group (n=60).”

Retention at each time point:
“Follow-up completion rates were high: 58 (96.7%) in the intervention group and 57 (95.0%) in the control group at 6 months and 54 (90.0%) and 56 (93.3%), respectively, at 9 months.”

Analysis population:
“All analyses were performed on an intention-to-treat basis using SPSS version 20.0…”

Denominators across use thresholds (intervention group only):

“Among intervention group participants, the frequency of message reading was analyzed as a marker of engagement. Patients were stratified into high (upper 50%) and low (lower 50%) message readers (Table 4). High readers accessed more than twice as many messages on average (130.5 vs. 50.6 messages, P<.001)...”

“At 9 months, this pattern persisted, with 71.4% of the improved group versus 30.3% of the non-improved group achieving ≥4 of 5 goals (p = 0.045, Supplemental Table 4).”

“Among 54 intervention participants who completed the satisfaction survey, 87.0% reported the messages to be easy to understand and helpful.”

16-ii) Primary analysis should be intent-to-treat

Primary analysis should be intent-to-treat, secondary analyses could include comparing only “users”, with the appropriate caveats that this is no longer a randomized sample (see 18-i).

	1	2	3	4	5	
subitem not at all important	○	○	○	○	○	essential

Does your paper address subitem 16-ii?

Copy and paste relevant sections from the manuscript (include quotes in quotation marks "like this" to indicate direct quotes from your manuscript), or elaborate on this item by providing additional information not in the ms, or briefly explain why the item is not applicable/relevant for your study

- Primary analysis approach:

“All analyses were performed on an intention-to-treat basis using SPSS version 20.0 (IBM Corp, Chicago, IL, USA).”

- Randomization and blinding description (relevant for understanding ITT approach):

“Participants were randomly assigned to either the intervention or control group using a centralized, computer-generated randomization table with a 1:1 allocation ratio…”

- Clarification that all randomized participants were analyzed according to their originally assigned group:

“Follow-up completion rates were high: 58 (96.7%) in the intervention group and 57 (95.0%) in the control group at 6 months and 54 (90.0%) and 56 (93.3%), respectively, at 9 months.”

- Secondary analyses based on usage (non-randomized comparisons):

- The manuscript also includes non-randomized exploratory analyses comparing “users” with higher vs. lower engagement:

“Among intervention group participants, the frequency of message reading was analyzed as a marker of engagement. Patients were stratified into high (upper 50%) and low (lower 50%) message readers (Table 4).”

“In a subgroup analysis, patients in the intervention group were categorized based on whether they demonstrated improvement in blood pressure by 9 months (n=23) or not (n=33)... Responders also read significantly more messages…”

17a) For each primary and secondary outcome, results for each group, and the estimated effect size and its precision (such as 95% confidence interval)

Does your paper address CONSORT subitem 17a? *

Copy and paste relevant sections from the manuscript (include quotes in quotation marks "like this" to indicate direct quotes from your manuscript), or elaborate on this item by providing additional information not in the ms, or briefly explain why the item is not applicable/relevant for your study

Primary outcome (blood pressure):

“Changes in blood pressure over time were not significantly different between groups at either 6 or 9 months (Figure 2; Table 2). At 9 months, mean systolic blood pressure was 126.0 mmHg in the intervention group vs. 128.0 mmHg in the control group (P=.943); diastolic pressure was 80.8 mmHg vs. 81.3 mmHg (P=.854).”

Secondary outcomes (e.g., lipid profile, HbA1c, BMI, smoking):

“Similarly, no significant differences were observed in secondary endpoints including lipid levels, HbA1c, BMI, walking distance, or smoking rates at either follow-up point.”

17a-i) Presentation of process outcomes such as metrics of use and intensity of use

In addition to primary/secondary (clinical) outcomes, the presentation of process outcomes such as metrics of use and intensity of use (dose, exposure) and their operational definitions is critical. This does not only refer to metrics of attrition (13-b) (often a binary variable), but also to more continuous exposure metrics such as “average session length”. These must be accompanied by a technical description how a metric like a “session” is defined (e.g., timeout after idle time) [1] (report under item 6a).

	1	2	3	4	5	
subitem not at all important	○	○	○	◉	○	essential

선택해제

Does your paper address subitem 17a-i?

Copy and paste relevant sections from the manuscript (include quotes in quotation marks "like this" to indicate direct quotes from your manuscript), or elaborate on this item by providing additional information not in the ms, or briefly explain why the item is not applicable/relevant for your study

- Definition of usage metrics:

The study defines “use” as message reading frequency and health diary input frequency:

“Among intervention group participants, the frequency of message reading was analyzed as a marker of engagement.”
“High readers accessed more than twice as many messages on average (130.5 vs. 50.6 messages, P<.001) and had significantly greater engagement in health diary input (250.2 vs. 46.8 entries, P=.001).” (Table 4)

- Operational definition of intensity:

Message exposure is quantified as “number of days messages were read” over the 6-month intervention:

“Participants in the intervention group received 6 messages per week over a 24-week period…”
“Messages were never repeated for the same individual.”
“Participants were stratified into high (upper 50%) and low (lower 50%) message readers.”

- Engagement outcome reporting:

These continuous process outcomes are analyzed in relation to clinical endpoints:

“Patients who read more messages (>median) showed greater health diary engagement and were more likely to meet ≥4 recommended CV risk factor targets at 9 months (69.0% vs. 19.2%, P <0.001).”

17b) For binary outcomes, presentation of both absolute and relative effect sizes is recommended

Does your paper address CONSORT subitem 17b? *

Copy and paste relevant sections from the manuscript (include quotes in quotation marks "like this" to indicate direct quotes from your manuscript), or elaborate on this item by providing additional information not in the ms, or briefly explain why the item is not applicable/relevant for your study

Binary outcomes such as achieving clinical targets (e.g., LDL-C <70 mg/dL, BP <140/90 mmHg, regular exercise, nonsmoking, BMI <25 kg/m^2) are reported in Table 3.

Example (from Table 3):

9th Month Outcome – LDL-C <70 mg/dL
“Intervention: 24/54 (44.4%), Control: 28/56 (50.0%)”
“Relative Risk: 0.89 (95% CI: 0.60–1.32), P = .573”

Achieving ≥4 of 5 guideline-recommended goals
“Intervention: 19/54 (35.2%), Control: 22/56 (39.3%)”
“Relative Risk: 0.90 (95% CI: 0.55–1.46), P = .697”

18) Results of any other analyses performed, including subgroup analyses and adjusted analyses, distinguishing pre-specified from exploratory

Does your paper address CONSORT subitem 18? *

Copy and paste relevant sections from the manuscript (include quotes in quotation marks "like this" to indicate direct quotes from your manuscript), or elaborate on this item by providing additional information not in the ms, or briefly explain why the item is not applicable/relevant for your study

- Exploratory subgroup analysis based on message engagement:

“Since the intervention using the AnSim application was message-mediated, the efficacy of the intervention could be dependent on participant message utilization. Among intervention group participants, the frequency of message reading was analyzed as a marker of engagement.”
“Patients were stratified into high (upper 50%) and low (lower 50%) message readers (Table 4). High readers accessed more than twice as many messages on average (130.5 vs. 50.6 messages, P<.001) and had significantly greater engagement in health diary input…”

- Exploratory subgroup analysis based on blood pressure improvement:

“In a subgroup analysis, patients in the intervention group were categorized based on whether they demonstrated improvement in blood pressure by 9 months (n=23) or not (n=33).”
“Responders also read significantly more messages (110.3 vs. 83.3 messages, P = .019) and achieved better overall CV risk factor control.” (Table 5)

- Distinguishing from main analysis:

These analyses are clearly secondary and post hoc, not part of the primary intention-to-treat analysis:

“Although no significant differences were observed in blood pressure or lipid levels between subgroups, a significantly larger proportion of high readers achieved ≥4 of 5 guideline-recommended goals…”

18-i) Subgroup analysis of comparing only users

A subgroup analysis of comparing only users is not uncommon in ehealth trials, but if done, it must be stressed that this is a self-selected sample and no longer an unbiased sample from a randomized trial (see 16-iii).

	1	2	3	4	5	
subitem not at all important	○	○	○	●	○	essential

선택해제

Does your paper address subitem 18-i?

Copy and paste relevant sections from the manuscript (include quotes in quotation marks "like this" to indicate direct quotes from your manuscript), or elaborate on this item by providing additional information not in the ms, or briefly explain why the item is not applicable/relevant for your study

- Subgroup analysis comparing only “users” (based on engagement):

“Among intervention group participants, the frequency of message reading was analyzed as a marker of engagement. Patients were stratified into high (upper 50%) and low (lower 50%) message readers (Table 4).”

“High readers accessed more than twice as many messages on average… and had significantly greater engagement in health diary input... A significantly larger proportion of high readers achieved ≥4 of 5 guideline-recommended goals at 9 months (69.0% vs. 19.2%, P <.001).”

- Also used in BP responder analysis:

“In a subgroup analysis, patients in the intervention group were categorized based on whether they demonstrated improvement in blood pressure by 9 months…”

19) All important harms or unintended effects in each group

(for specific guidance see CONSORT for harms)

Does your paper address CONSORT subitem 19? *

Copy and paste relevant sections from the manuscript (include quotes in quotation marks "like this" to indicate direct quotes from your manuscript), or elaborate on this item by providing additional information not in the ms, or briefly explain why the item is not applicable/relevant for your study

In the “Clinical Events” subsection of the Results:

“During the 9-month follow-up, 2 clinical events occurred, both in the control group (1 coronary revascularization and 2 readmissions), but the difference between groups was not significant (P=.496; Supplemental Table 3). No deaths, myocardial infarctions, or heart failure hospitalizations were reported in either group.”

19-i) Include privacy breaches, technical problems

Include privacy breaches, technical problems. This does not only include physical “harm” to participants, but also incidents such as perceived or real privacy breaches [1], technical problems, and other unexpected/unintended incidents. “Unintended effects” also includes unintended positive effects [2].

	1	2	3	4	5	
subitem not at all important	○	○	◉	○	○	essential

선택해제

Does your paper address subitem 19-i?

Copy and paste relevant sections from the manuscript (include quotes in quotation marks "like this" to indicate direct quotes from your manuscript), or elaborate on this item by providing additional information not in the ms, or briefly explain why the item is not applicable/relevant for your study

“Among 54 intervention participants who completed the satisfaction survey, 87.0% reported the messages to be easy to understand and helpful. A majority (81.4%) expressed a desire to continue receiving messages, and 87.0% would recommend the app to others.”

and we can add-on privacy and data security & technical stability of the app part in the manuscript as below (which were already mentioned above);

“Data privacy was ensured through encryption and secure servers, and participants were provided with contact details to report any concerns.”

“No changes in app functionality, message content, or technical downtimes occurred during the intervention period.”

19-ii) Include qualitative feedback from participants or observations from staff/researchers

Include qualitative feedback from participants or observations from staff/researchers, if available, on strengths and shortcomings of the application, especially if they point to unintended/unexpected effects or uses. This includes (if available) reasons for why people did or did not use the application as intended by the developers.

	1	2	3	4	5	
subitem not at all important	○	○	○	○	○	essential

Does your paper address subitem 19-ii?

Copy and paste relevant sections from the manuscript (include quotes in quotation marks "like this" to indicate direct quotes from your manuscript), or elaborate on this item by providing additional information not in the ms, or briefly explain why the item is not applicable/relevant for your study

- Focus group interview during development (from Methods > Message Development):

“To capture patient needs and inform message development, a focus group interview was conducted with 8 patients who had previously undergone PCI and used a smartphone… The subject of the interview consisted of 5 categories: 1) degree of utilization of the smartphone application, 2) exercise, 3) nutrition, 4) stress management, and 5) knowledge about CAD and prevention.”

“Interviews were recorded with patient consent and textualized in verbatim form, and qualitative analysis was performed using NVivo.”

- User satisfaction survey results (from Results > App Acceptability):

“Among 54 intervention participants who completed the satisfaction survey, 87.0% reported the messages to be easy to understand and helpful. A majority (81.4%) expressed a desire to continue receiving messages, and 87.0% would recommend the app to others.”

- Education level and engagement barrier:

“Message reading frequency differed by education level, with elementary school graduates reading significantly fewer messages (P=.045; Supplemental Table 5).”

DISCUSSION

22) Interpretation consistent with results, balancing benefits and harms, and considering other relevant evidence

NPT: In addition, take into account the choice of the comparator, lack of or partial blinding, and unequal expertise of care providers or centers in each group

22-i) Restate study questions and summarize the answers suggested by the data, starting with primary outcomes and process outcomes (use)

Restate study questions and summarize the answers suggested by the data, starting with primary outcomes and process outcomes (use).

	1	2	3	4	5	
subitem not at all important	○	○	○	○	◉	essential

선택해제

Does your paper address subitem 22-i? *

Copy and paste relevant sections from the manuscript (include quotes in quotation marks "like this" to indicate direct quotes from your manuscript), or elaborate on this item by providing additional information not in the ms, or briefly explain why the item is not applicable/relevant for your study

- Restating study question:

"In this single-blinded randomized controlled trial, we evaluated the effectiveness of a patient-specific, smartphone-based messaging application (AnSim) on CV risk factor control in patients following PCI."

- Summary of primary outcome:

"While the primary endpoint—change in blood pressure—did not differ significantly between the intervention and control groups over 9 months…"

- Summary of process outcomes (app use and engagement):

"…participants with higher message engagement (top 50%) achieved significantly better adherence to guideline-recommended targets for LDL-C, blood pressure, and lifestyle modification (e.g., exercise, smoking cessation)."
"Individuals who experienced meaningful blood pressure reductions by 9 months had higher baseline BP, read more messages, and exhibited greater behavior change over time…"

- Interpretation linked to data and consistent with results:

"These findings suggest that mobile health interventions may be most effective in motivated individuals and highlight the importance of tailoring content and enhancing user engagement."

22-ii) Highlight unanswered new questions, suggest future research

Highlight unanswered new questions, suggest future research.

	1	2	3	4	5	
subitem not at all important	○	○	○	○	◉	essential

선택해제

Does your paper address subitem 22-ii?

Copy and paste relevant sections from the manuscript (include quotes in quotation marks "like this" to indicate direct quotes from your manuscript), or elaborate on this item by providing additional information not in the ms, or briefly explain why the item is not applicable/relevant for your study

- Identification of limitations and need for future studies:

"However, limitations include a modest sample size and short follow-up period, limiting power to detect differences in long-term CV outcomes."

- Call for future studies with longer follow-up and larger samples:

"Larger trials with longer follow-up are needed to determine whether these interventions improve clinical outcomes such as hospital readmissions and mortality."

- Proposal for comparative effectiveness research:

"Comparative effectiveness studies between full-scale digital rehabilitation programs and lighter interventions like AnSim will also help clarify the optimal design and intensity of mobile CR."

- Suggestion for integration into broader healthcare systems:

"Future versions of AnSim should incorporate richer multimedia, user-driven goal setting, and 2-way communication. Moreover, integrating app data with EMRs and care teams could facilitate more proactive medical adjustments..."

- Equity and accessibility implications:

"While age did not impact message engagement, education level appeared to be a determinant… Future interventions should incorporate more intuitive interfaces, visual content, and adaptive education levels to improve inclusivity and reduce disparities."

20) Trial limitations, addressing sources of potential bias, imprecision, and, if relevant, multiplicity of analyses

20-i) Typical limitations in ehealth trials

Typical limitations in ehealth trials: Participants in ehealth trials are rarely blinded. Ehealth trials often look at a multiplicity of outcomes, increasing risk for a Type I error. Discuss biases due to non-use of the intervention/usability issues, biases through informed consent procedures, unexpected events.

	1	2	3	4	5	
subitem not at all important	○	○	○	○	◉	essential

선택해제

Does your paper address subitem 20-i? *

Copy and paste relevant sections from the manuscript (include quotes in quotation marks "like this" to indicate direct quotes from your manuscript), or elaborate on this item by providing additional information not in the ms, or briefly explain why the item is not applicable/relevant for your study

- Lack of blinding:

“Due to the nature of the intervention, which involved receiving personalized smartphone messages, participants were not blinded after group allocation.”

- Acknowledgment of limitations in design and generalizability:

“Limitations include a modest sample size and short follow-up period, limiting power to detect differences in long-term CV outcomes.”
“Additionally, our intervention lacked multimedia interactivity, real-time feedback, or structured exercise modules, which may have reduced efficacy.”

- Acknowledgment of digital engagement limitations:

“App usage data from the control group were unavailable, preventing direct comparisons of digital engagement.”
“Education level appeared to be a determinant: participants with only elementary education read fewer messages… Future interventions should incorporate more intuitive interfaces, visual content, and adaptive education levels…”

- Discussion of post-randomization use/non-use bias:

“Our results demonstrate that engagement—not mere exposure—is a primary driver of digital intervention efficacy.”

21) Generalisability (external validity, applicability) of the trial findings

NPT: External validity of the trial findings according to the intervention, comparators, patients, and care providers or centers involved in the trial

21-i) Generalizability to other populations

Generalizability to other populations: In particular, discuss generalizability to a general Internet population, outside of a RCT setting, and general patient population, including applicability of the study results for other organizations

	1	2	3	4	5	
subitem not at all important	○	○	○	◉	○	essential

선택해제

Does your paper address subitem 21-i?

Copy and paste relevant sections from the manuscript (include quotes in quotation marks "like this" to indicate direct quotes from your manuscript), or elaborate on this item by providing additional information not in the ms, or briefly explain why the item is not applicable/relevant for your study

- Indication of the trial setting and sample characteristics:

“The trial was conducted at 2 sites in Korea: a secondary general hospital (Sejong General Hospital) and a tertiary academic hospital (Korea University Guro Hospital).”
“The mean age of participants was 58.5 years, and 84.2% were male.”

- Recognition of subgroup limitations (e.g., low-literacy users):

“Education level appeared to be a determinant: participants with only elementary education read fewer messages… Future interventions should incorporate more intuitive interfaces, visual content, and adaptive education levels to improve inclusivity and reduce disparities.”

21-ii) Discuss if there were elements in the RCT that would be different in a routine application setting

Discuss if there were elements in the RCT that would be different in a routine application setting (e.g., prompts/reminders, more human involvement, training sessions or other co-interventions) and what impact the omission of these elements could have on use, adoption, or outcomes if the intervention is applied outside of a RCT setting.

	1	2	3	4	5	
subitem not at all important	○	◉	○	○	○	essential

선택해제

Does your paper address subitem 21-ii?

Copy and paste relevant sections from the manuscript (include quotes in quotation marks "like this" to indicate direct quotes from your manuscript), or elaborate on this item by providing additional information not in the ms, or briefly explain why the item is not applicable/relevant for your study

“All participants received brief onboarding at enrollment.”
“Participants in the intervention group also received weekly feedback messages based on their logged health data, sent by a designated healthcare provider.”

OTHER INFORMATION

23) Registration number and name of trial registry

Does your paper address CONSORT subitem 23? *

Copy and paste relevant sections from the manuscript (include quotes in quotation marks "like this" to indicate direct quotes from your manuscript), or elaborate on this item by providing additional information not in the ms, or briefly explain why the item is not applicable/relevant for your study

“Trial Registration: Clinical Research Information Service (CRIS) KCT0002361; https://cris.nih.go.kr/cris”

24) Where the full trial protocol can be accessed, if available

Does your paper address CONSORT subitem 24? *

Cite a Multimedia Appendix, other reference, or copy and paste relevant sections from the manuscript (include quotes in quotation marks "like this" to indicate direct quotes from your manuscript), or elaborate on this item by providing additional information not in the ms, or briefly explain why the item is not applicable/relevant for your study

“Trial Registration: Clinical Research Information Service (CRIS) KCT0002361; https://cris.nih.go.kr/cris”

25) Sources of funding and other support (such as supply of drugs), role of funders

Does your paper address CONSORT subitem 25? *

Copy and paste relevant sections from the manuscript (include quotes in quotation marks "like this" to indicate direct quotes from your manuscript), or elaborate on this item by providing additional information not in the ms, or briefly explain why the item is not applicable/relevant for your study

“This research was supported by a grant from the Korea Health Industry Development Institute (KHIDI), funded by the Ministry of Health and Welfare, Republic of Korea (grant number: HI16C0483). The funders had no role in the design of the study, data collection and analysis, interpretation of data, or writing of the manuscript.”

X27) Conflicts of Interest (not a CONSORT item)

X27-i) State the relation of the study team towards the system being evaluated

In addition to the usual declaration of interests (financial or otherwise), also state the relation of the study team towards the system being evaluated, i.e., state if the authors/evaluators are distinct from or identical with the developers/sponsors of the intervention.

	1	2	3	4	5	
subitem not at all important	○	○	◉	○	○	essential

선택해제

Does your paper address subitem X27-i?

Copy and paste relevant sections from the manuscript (include quotes in quotation marks "like this" to indicate direct quotes from your manuscript), or elaborate on this item by providing additional information not in the ms, or briefly explain why the item is not applicable/relevant for your study

- The app evaluated (AnSim) was developed by a collaborative team that includes some study authors, as described under "Message Development":

"A multidisciplinary team—including cardiologists, behavioral scientists, and app developers—collaborated on content creation."

- Author affiliations include:

Hanmi Healthcare, which is listed in the author list as an institutional affiliation:

- In the Acknowledgments:

"We gratefully acknowledge the contributions of Yeonghun Song and Gyeongho Jung from the IT business division at Hanmi Healthcare for their work in application development and study operations."

About the CONSORT EHEALTH checklist

As a result of using this checklist, did you make changes in your manuscript? *

- [ ] yes, major changes
- [x] yes, minor changes
- [ ] no

What were the most important changes you made as a result of using this checklist?

It seems to be able to add additional contents by confirming that specific parts are missing, especially in the method section.

How much time did you spend on going through the checklist INCLUDING making changes in your manuscript *

I spend my times for about 12 hours

As a result of using this checklist, do you think your manuscript has improved? *

- [x] yes
- [ ] no
- [ ] 기타:

Would you like to become involved in the CONSORT EHEALTH group?

This would involve for example becoming involved in participating in a workshop and writing an "Explanation and Elaboration" document

- ○ yes
- ◉ no
- ○ 기타:

선택해제

Any other comments or questions on CONSORT EHEALTH

This manuscript was carefully prepared in accordance with the CONSORT-eHEALTH checklist. Considerable effort was invested to ensure that all relevant CONSORT subitems were explicitly addressed, enhancing transparency and completeness of reporting. However, due to the extensive nature of the CONSORT-eHEALTH guidelines, we acknowledge that not every subitem may be applicable or fully elaborated upon within the manuscript.

STOP - Save this form as PDF before you click submit

To generate a record that you filled in this form, we recommend to generate a PDF of this page (on a Mac, simply select "print" and then select "print as PDF") before you submit it.

When you submit your (revised) paper to JMIR, please upload the PDF as supplementary file.

Don't worry if some text in the textboxes is cut off, as we still have the complete information in our database. Thank you!

Final step: Click submit !

Click submit so we have your answers in our database!

제출 양식 지우기

Google Forms를 통해 비밀번호를 제출하지 마세요.

이 콘텐츠는 Google이 만들거나 승인하지 않았습니다. - 양식 소유자에게 문의 - 서비스 약관 - 개인정보처리방침

양식이 의심스러운가요? 보고서

Google 설문지
